# Uncovering Unique Green Algae and Cyanobacteria Isolated from Biocrusts in Highly Saline Potash Tailing Pile Habitats, Using an Integrative Approach

**DOI:** 10.3390/microorganisms8111667

**Published:** 2020-10-27

**Authors:** Veronika Sommer, Tatiana Mikhailyuk, Karin Glaser, Ulf Karsten

**Affiliations:** 1 Institute for Biological Sciences, Applied Ecology and Phycology, University of Rostock, 18059 Rostock, Germany; veronika.sommer@uni-rostock.de (V.S.); karin.glaser@uni-rostock.de (K.G.); 2 upi UmweltProjekt Ingenieursgesellschaft mbH, 39576 Stendal, Germany; 3 National Academy of Sciences of Ukraine, M.G. Kholodny Institute of Botany, 01601 Kyiv, Ukraine; t-mikhailyuk@ukr.net

**Keywords:** biocrust, salinity, potash mining, microalgae, cyanobacteria, integrative approach

## Abstract

Potash tailing piles caused by fertilizer production shape their surroundings because of the associated salt impact. A previous study in these environments addressed the functional community “biocrust” comprising various micro- and macro-organisms inhabiting the soil surface. In that previous study, biocrust microalgae and cyanobacteria were isolated and morphologically identified amongst an ecological discussion. However, morphological species identification maybe is difficult because of phenotypic plasticity, which might lead to misidentifications. The present study revisited the earlier species list using an integrative approach, including molecular methods. Seventy-six strains were sequenced using the markers small subunit (SSU) rRNA gene and internal transcribed spacer (ITS). Phylogenetic analyses confirmed some morphologically identified species. However, several other strains could only be identified at the genus level. This indicates a high proportion of possibly unknown taxa, underlined by the low congruence of the previous morphological identifications to our results. In general, the integrative approach resulted in more precise species identifications and should be considered as an extension of the previous morphological species list. The majority of taxa found were common in saline habitats, whereas some were more likely to occur in nonsaline environments. Consequently, biocrusts in saline environments of potash tailing piles contain unique microalgae and cyanobacteria that will possibly reveal several new taxa in more detailed future studies and, hence, provide new data on the biodiversity, as well as new candidates for applied research.

## 1. Introduction

Biological soil crusts (biocrusts) are multidimensional communities consisting of various micro- and macro-organisms, which inhabit the first millimeters of the soil [1]. They include microalgae and cyanobacteria, as well as protists, heterotrophic bacteria, and fungi, and in later successional stages, also lichens and mosses might grow [1]. As ecosystem engineers, biocrusts increase the soil stability; alter hydrological cycles; and provide nutrients via primary production, aerial N-fixation, dust trapping, and bio-weathering [2,3]. Biocrusts occur in all climate zones worldwide; they have their greatest impacts where vascular plant growth is limited due to extreme abiotic stressors [2]. In drylands, biocrusts cover large areas, thus creating biodiversity hot spots in these hostile environments [2], and can reach a stable climax state [4]. However, biocrusts are vulnerable against disturbances, such as trampling [5,6,7,8], and endangered by climate change [9]. This stresses the relevance of biocrust research, along with the great interest on their multifunctional ecosystem services in the growing field of applied biocrust research [10,11,12,13,14].

Microalgae and cyanobacteria in biocrusts are well-adapted to their harsh, mainly arid environments [15]. Cyanobacteria and filamentous algae are key organisms in biocrusts and excrete sticky extracellular polysaccharides (EPS). EPS are important in promoting the cohesion of biocrust inhabitants and the connection to soil particles and act as water-holding biomolecules in drought tolerance [16]. Overall, desiccation stress in biocrusts has been intensively studied, with the focus on a variety of organisms such as green algae [17,18] and cyanobacteria [19,20] to address the large-scale ecological aspects [21,22], as well as the (eco-)physiological processes [23,24].

Similarly, the salt tolerance of cyanobacteria [25,26], as well as of algae, in marine habitats [27,28] is well-studied. Cells under saline conditions are exposed to water deprivation analogous to drought conditions but, also, show differences in some physiological processes [25,28]. In contrast to the total absence of water, salt-stressed cells can still be in contact with liquid water yet be subjected to a diminished water potential. The loss of intracellular water due to the high osmotic potential outside the cell can be hindered by inorganic and organic osmolytes. These compatible solutes increase the osmotic potential within the cell and thereby prevent water loss. In addition, the toxic Na^+^ ions are exchanged for the nutrient K^+^ to maintain the cellular ion balance. Due to their increased production of some commercially relevant secondary metabolites under salt stress, certain microalgae such as *Dunaliella salina* are of interest for large-scale biomass production [29,30]. Hypersaline wastewater from the potash industry can be used for this purpose [31].

Halophilic microbial communities have been addressed in a variety of studies, such as microbial mats in hypersaline lakes [32,33,34] and lagoonal mats [35,36], as well as benthic communities in solar saltern plants [37,38,39]. In these hypersaline aquatic habitats, photosynthetic organisms were found in salinities even higher than 30%, such as the green alga *Dunaliella* [40,41] and the cyanobacteria *Geitlerinema*, *Phormidium* and *Komvophoron* [42]. Further, several studies have described the biodiversity of algae and cyanobacteria in saline terrestrial habitats [35,42,43,44,45,46,47,48,49,50,51,52,53]. Most of them focused on natural saline habitats.

Anthropogenic salinization, however, is increasingly affecting aquatic [54], as well as terrestrial [55,56], ecosystems on a global scale. In Europe, one of the main drivers of anthropogenic salinization is the potash industry for fertilizer production [57]. The valuable components (KCl and MgSO_4_) of the mined potash salt are separated from the residue NaCl. Potash tailing piles, as a consequence, consist of highly saline overburden (mostly of NaCl). In central Germany, in particular, potash tailing piles, reaching a height of 200 m, shape the landscape. Rainfall dissolves the deposited salt and washes out highly saline pile wastewaters, which, in one potash tailing pile in Germany, contained dissolved concentrations up to 320 g/L (very close to saturation; predominantly Na^+^ and Cl^−^) [58]. Without a base seal, this process leads to a salinization of the surrounding ecosystems.

As a result, unique salt-affected ecosystems emerge, inhabited by adapted costal flora and fauna, despite a distance of about 400 km inland from the coast. Halophyte plant communities, with members such as the sea aster (*Tripolium pannonicum*) and glasswort (*Salicornia* sp.), have been well-studied close to these tailing piles [59,60,61]. However, higher plants occur only in the surroundings of potash tailing piles and do not inhabit the pile body itself, due to the extreme salinity of the overburden. Little is known about biocrusts in the surroundings of such hypersaline potash tailing piles. Eilmus et al. [62] found a rich prokaryotic community directly on the bare material of a German potash tailing pile, whereas our previous study described diverse communities of both cyanobacteria and eukaryotic microalgae in biological soil crusts between the less-saline vegetation line and the hyper-saline bare pile bodies of several potash tailing piles across Germany [63]. In this previous study, we found species numbers comparable to those in less-saline regions, which underlined the significance of research on those specific terrestrial habitats. However, that study was based on morphological observations only. Investigations on biocrust algae in saline terrestrial inland habitats using modern molecular phylogenetic approaches are still rare.

Morphological methods based on light microscopy have a long tradition and continue to be essential for microalgal and cyanobacteria species identification. However, the phenotypic plasticity of unicellular algae due to culture conditions, age, and reproductive stages complicate identifications. In particular, salinity has an impact on the morphology of algal cells [64]. In addition, microalgal and cyanobacteria cells often lack distinct morphological traits, which limits the use of light microscopy for accurate species identification. This limitation often results in a high proportion of identifications only to the family or genus level [65]. Sommer et al. [63], for example, identified only 32% of their microalgal strains from saline potash tailing piles to the species level.

To accurately identify algal strains, an integrative approach should be considered. This approach combines classical morphological methods with modern molecular phylogenetic analyses. It was introduced by Komárek [66] for cyanobacteria, followed by Pröschold and Leliaert [67] for green algae, and has been increasingly used in microalgal [65,68,69,70,71,72,73], as well as cyanobacterial [35,74,75,76,77], research. Some recent reviews stressed the advantages and challenges of this approach [78,79,80,81]. Still, some limitations on microalgal and cyanobacteria identification remain, such as a lack of identified sequences in databases; nevertheless, the combination of the mentioned methods highly improves the quality of the species lists, thereby providing precise phylogenetic, and not biodiversity, information.

Revisiting morphologically identified strains with molecular methods can lead to the finding of new species or rare taxa that would have been overlooked by using only light microscopy [65,72,82]. Particularly in extreme habitats, the probability of finding yet undiscovered species is high [83] due to the unique ecological niches inhabited by adapted organisms. In the extreme habitats of potash tailing piles, there might well be a high potential of discovering previously unknown, highly specialized cyanobacteria and microalgae.

The aim of our study was to complement the information on biocrust microalgal and cyanobacterial cultures from the surroundings of saline potash tailing piles as a continuation of the previous morphological observations [63]. Therefore, common conserved markers for the small subunit ribosomal ribonucleic acid (SSU rRNA) gene, as well as the variable internal transcribed spacer (ITS), were used to elucidate the identity and phylogenetic classification of these green algae and cyanobacteria. Using the integrative approach, we assumed that we would be able to generate more detailed phylogenetic information compared to the morphological observations alone.

## 2. Materials and Methods

### 2.1. Algal and Cyanobacteria Isolates and Their Maintenance

**Figure 1:**
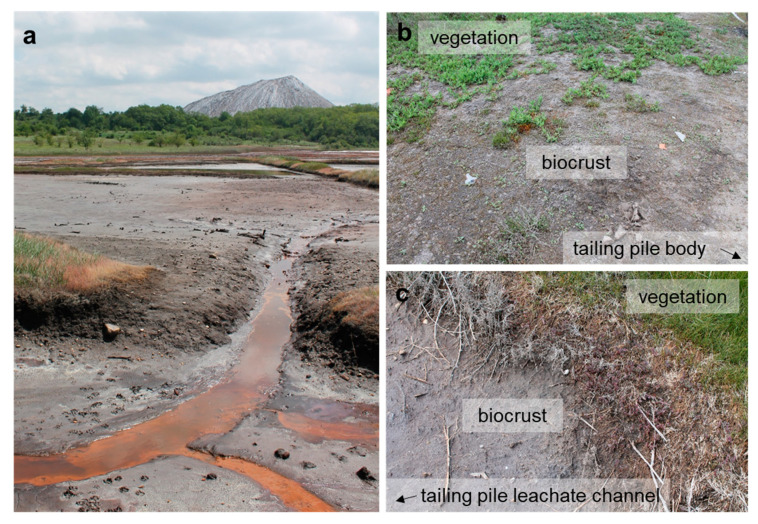
The potash tailing pile in Teutschenthal, Germany. (**a**) The plain in front of the tailing pile represents an abandoned lignite mine, which is regularly flooded by highly saline tailing pile leachate. (**b**) Biocrusts close to the Teutschenthal tailing pile between the vegetation and tailing pile body and (**c**) between the vegetation and tailing pile leachate water channels.

In total, 76 original strains were analyzed in this study. A total of 66 strains, including 52 green algae and 14 cyanobacteria strains isolated from the surroundings of potash tailing piles in Germany, were published earlier. This paper included the respective site descriptions, samplings, and isolation procedures [63]. In addition, ten unpublished green algae strains isolated from two of the sampling sites (TT and NN) of the previous publication [63], as well as from another location at the Teutschenthal tailing piles (TTF, Figure 1), were included in this study. The latter site was situated in the nearest environment to the tailing piles in an abandoned lignite mine (Figure 1). Rainwater dissolves some salt from the tailing pile. These strongly saline brines frequently flood the former mining area. Thin biocrusts were found between the brine channels and the bordering vegetation (Figure 1c). The sampling procedure and isolation of the unialgal strains were performed as described previously [63].

Unialgal cultures were maintained on solidified 3N-BBM+V [84] and cyanobacteria cultures on BG-11 [85], both media enriched with 3% NaCl. Cultures were kept at 20 °C with a light/dark cycle of 16:8 h with 30-µm photons m^−2^ s^−1^ (Osram Lumilux Cool White lamps L36W/840, Munich, Germany). Isolates were morphologically evaluated using an Olympus BX51 light microscope with Nomarski DIC optics (Olympus Ltd., Hamburg, Germany), and photomicrographs were taken with the digital camera Olympus UC30 attached to the microscope and processed with the cellSense Entry imaging software (v. 2.1, Olympus Soft Imaging Solutions, Münster, Germany). The morphological species list of green algae and cyanobacteria isolates from potash tailing pile areas was published by Sommer et al. [63].

### 2.2. DNA Extraction, PCR, and Sequencing

The SSU region, as well as the ITS1-5.8S-ITS2 region (hereafter, ITS-1,2 regions), and in particular groups, the ITS-2 region for green algae and the SSU-LSU rRNA (large subunit rRNA) intergenic spacer for cyanobacteria were used as molecular markers. The cells of green algae and cyanobacteria were disrupted by shaking with 1.25–1.55-mm glass beads in combination with a threefold freezing and thawing cycle (liquid N, heating block 65 °C). The DNA was extracted with the NucleoSpin Plant II mini kit (Macherey Nagel, Düren, Germany) following the instructions. PCR was performed as described by Mikhailyuk et al. [86] using the primers EAF3 and ITS055R for green algae [87,88] and SSU-4-forw and ptLSU C-D-rev for cyanobacteria [89]. Sanger sequencing was conducted by GATC Sequencing Services (Eurofins Genomics Germany, Ebersberg, Germany) using the primers EAF3 and 1400R [87], N920R [88], 536R [90], 920F and 1400F [91], and GF and GR [92] for green algae and SSU-4-forw, Wil 6, Wil 12, Wil 14, Wil 5, Wil 9, Wil 16, and ptLSU C-D-rev [89,93] for cyanobacteria.

### 2.3. Phylogenetic Analyses

Bioinformatics steps were performed using the software Geneious 8.1.9 (Biomatters Ltd., v. 8.1.9, Auckland, New Zealand), if not stated differently. The sequences of the isolates were compared to identified taxa in the Gene Bank database using BLASTn [94]. Top BLAST hits, as well as some additional sequences, were downloaded (Supplementary Table S1) and aligned with the original isolates. The MUSCLE algorithm [95] was used with a maximum of eight iterations. The alignments were cut and checked for errors manually. Bayesian phylogenic trees were calculated with the MrBayes [96] add-in by using the GTR+G+I evolutionary model run for 5,000,000 generations and taking the trees every 500 generations. Of the four runs of the Markov chain Monte Carlo, two were run separately, with split frequencies at the end of the runs below 0.01. Maximum-likelihood (ML) analyses were performed to verify the Bayesian results, using GARLI [97] with 1000 bootstrap replicates. Phylogenetic consensus trees were edited in the MEGA-X 10.1 software [98] and finalized with PowerPoint (Microsoft Office, Standard 2013, Redmond, WA, USA).

Taxa were named after current taxonomically accepted species or genus names stated in AlgaeBase [99] using the respective references (Table 1). Subsequently, the original habitats of all strains were classified as follows: aquatic (frequent presence of water, e.g., marine, freshwater, brackish water, bog water; both planktonic and benthic, or in microbial mats); terrestrial (in the absence of permanent water, e.g., on soil, bark, leaves, stones, facades, in biocrusts, or other nonaquatic biofilms); and endophytic/phycobiontic (e.g., phycobionts in lichens or endophytes in macrophytes, hereafter endophytic). The symbols are colored according to the salinity of the habitats (blue = nonsaline, e.g., freshwater and soil; orange = saline, e.g., marine, brackish and saline soil; black = other extreme conditions, e.g., acidity, heat, and radioactivity; and grey: unknown).

## 3. Results

### 3.1. SSU rRNA Gene Phylogeny

**Figure 2:**
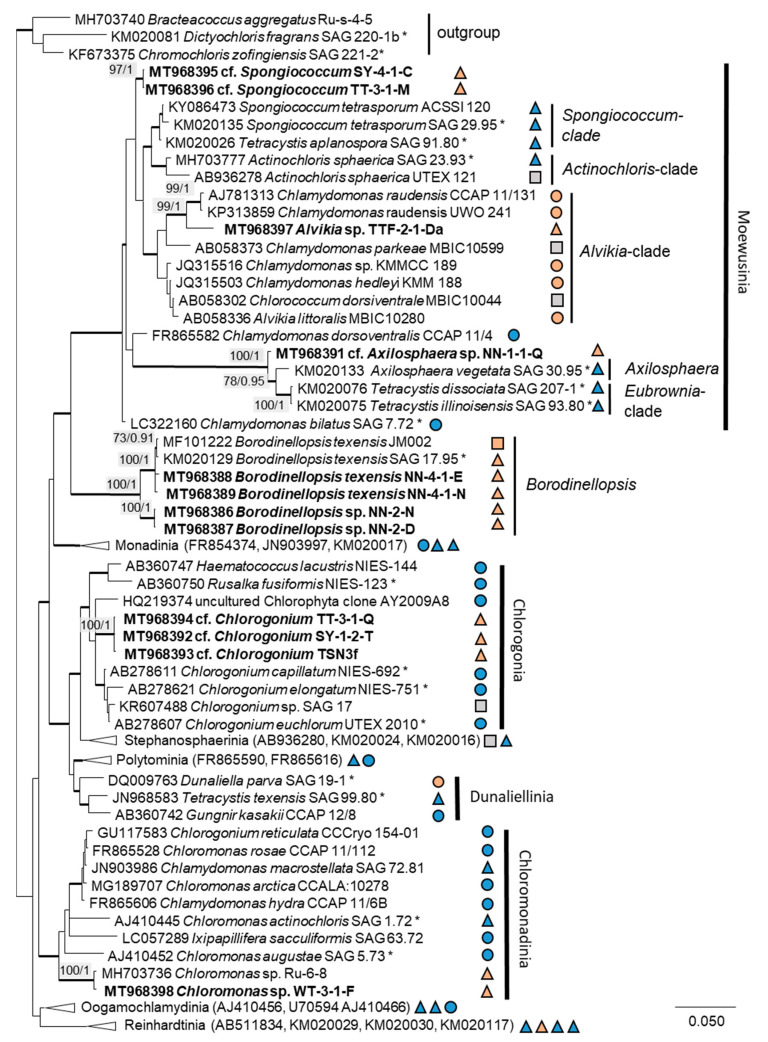
Molecular phylogeny of the Chlamydomonadales based on small subunit ribosomal ribonucleic acid (SSU rRNA) gene sequencing. The phylogenetic tree was calculated by the Bayesian method, including posterior probabilities (PP) with additional maximum likelihood bootstrap values (BP); branches are supported by both methods in bold (BP > 60% and PP > 0.9). Authentic strains were marked with an asterisk, original strains from this study in bold. Habitats of the strains were classified according to habitat type (Δ terrestrial = absence of permanent water, o aquatic = presence of water, and □ unknown) and saline (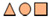); nonsaline (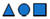); and unknown (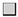) habitats.

**Figure 3:**
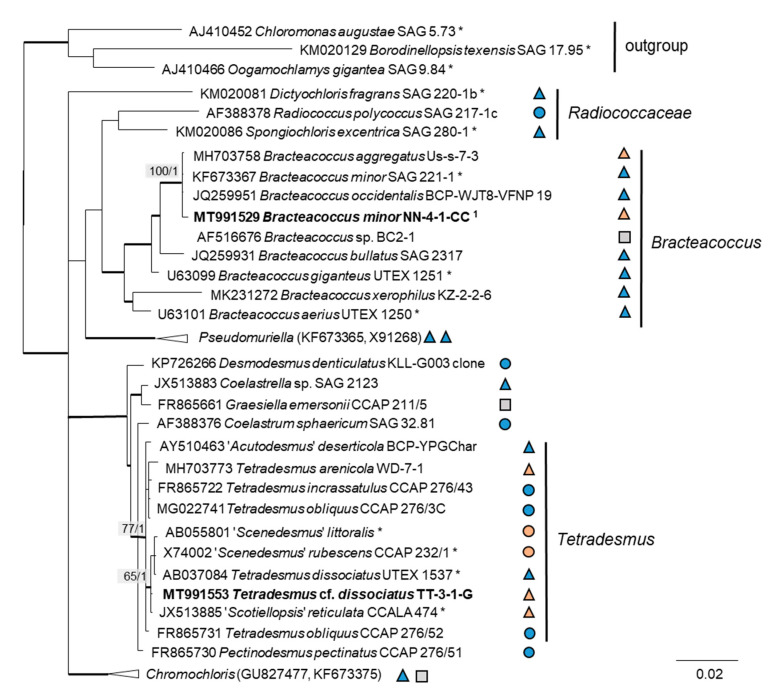
Molecular phylogeny of the Sphaeropleales based on SSU rRNA gene sequencing. The phylogenetic tree was calculated by the Bayesian method, including posterior probabilities (PP) with additional maximum likelihood bootstrap values (BP); branches are supported by both methods in bold (BP > 60% and PP > 0.9). Authentic strains were marked with an asterisk, original strains from this study in bold. Habitats of the strains were classified according to habitat type (Δ terrestrial = absence of permanent water, o aquatic = presence of water, and □ unknown) and saline (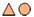); nonsaline (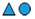); and unknown (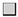) habitats. ^1^ Identical to *Bracteacoccus minor* NN-4-1-CC (MT991529), NN-4-1-H (MT991530), NN-4-1-D2 (MT968390), TTF-1-1-M (MT991533), TTF-2-1-A (MT991534), and TTF-2-1-J (MT991535).

The 76 original strains were distributed among the Chlorophyta (62) and cyanobacteria (14). All green-algal strains belonged to the phylum Chlorophyta; the phylum Streptophyta was absent. Of the Chlorophyta, 32% (20 isolates) fell into the Chlorophyceae. The majority of these original strains (11 isolates) were placed in the order Chlamydomonadales (Figure 2). We found lineages in the Moewusinia (genus *Alvikia* and two separate lineages close to the genera *Spongiococcum*/*Actinochloris* and *Axilosphaera*/*Eubrownia*), Chlorogonia (separate lineage close to *Chlorogonium*), and Chloromonadinia (*Chloromonas*), as well as an independent lineage formed by the genus *Borodinellopsis*. In the second order present, the Sphaeropleales (nine isolates, 14%; Figure 3), the strains clustered in the lineages formed by the genera *Bracteacoccus* and *Tetradesmus*.

**Figure 4:**
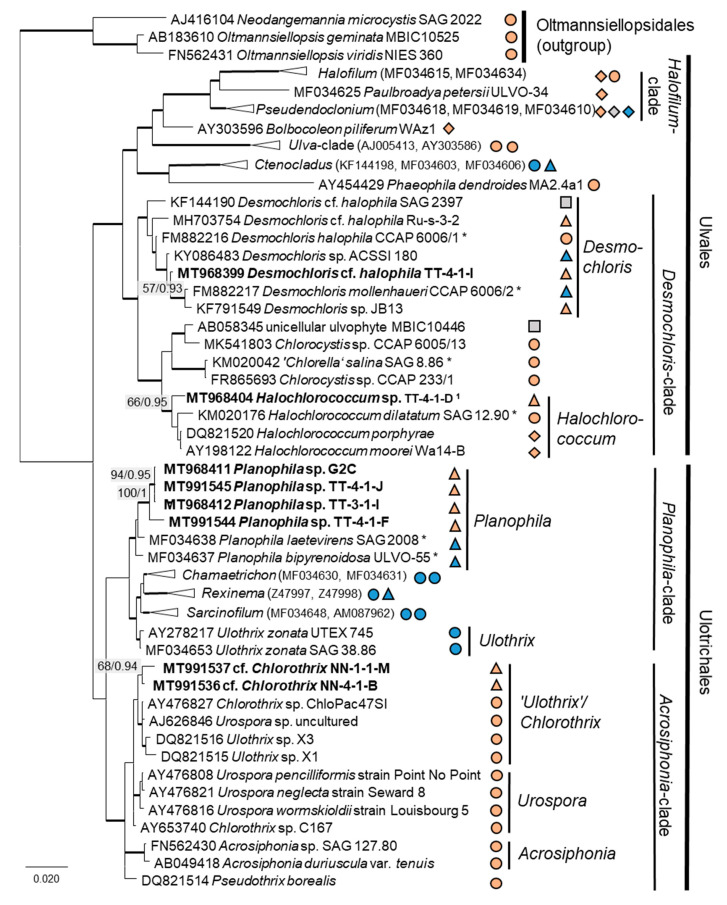
Molecular phylogeny of the Ulvophyceae based on SSU rRNA gene sequencing. The phylogenetic tree was calculated by the Bayesian method, including posterior probabilities (PP) with additional maximum likelihood bootstrap values (BP); branches are supported by both methods in bold (BP > 60% and PP > 0.9). Authentic strains were marked with an asterisk, original strains from this study in bold. Habitats of the strains were classified according to habitat type (Δ terrestrial = absence of permanent water, o aquatic = presence of water, ◊ phycobiontic or endophytic, and □ unknown) and saline (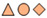); nonsaline (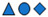), and unknown (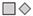) habitats. ^1^ Identical to *Halochlorococcum* sp. TT-4-1-M (MT968405), TT-4-1-O (MT968406), NN-4-1-Q (MT968401), NN-4-1-S (MT968402), and NN-4-1-T (MT968403).

**Figure 5:**
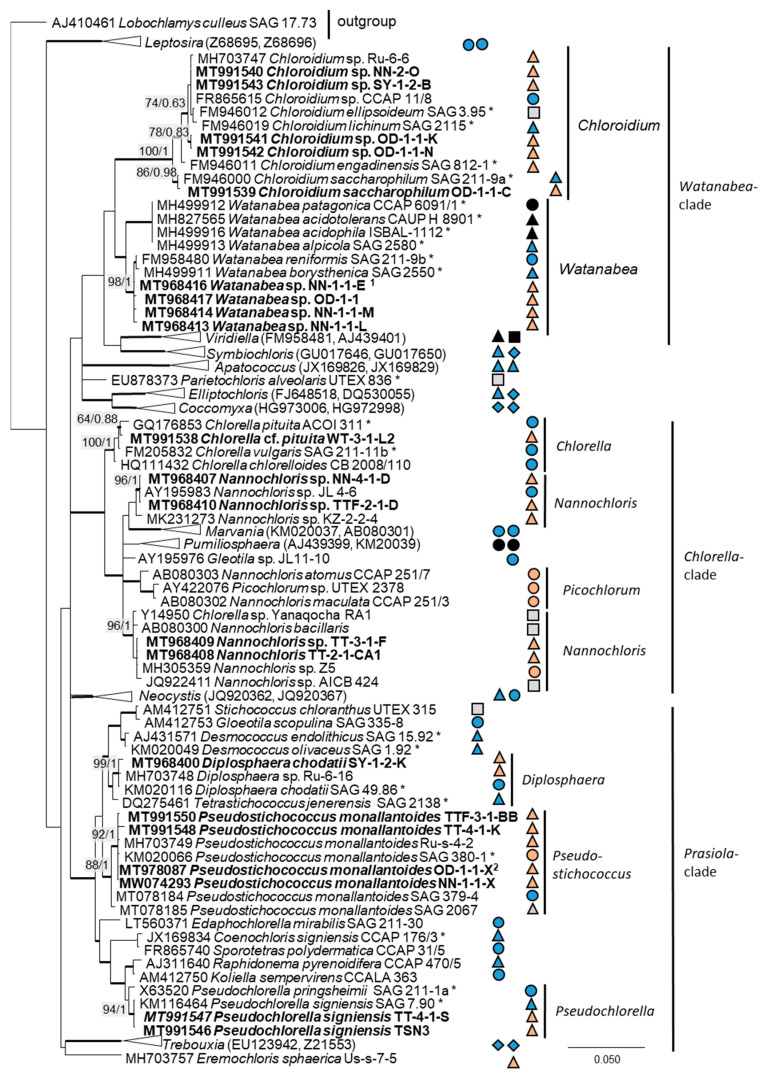
Molecular phylogeny of the Trebouxiophyceae based on SSU rRNA gene sequencing. The phylogenetic tree was calculated by the Bayesian method, including posterior probabilities (PP) with additional maximum likelihood bootstrap values (BP); branches are supported by both methods in bold (BP > 60% and PP > 0.9). Authentic strains were marked with an asterisk, original strains from this study in bold. Habitats of the strains were classified according to habitat type (Δ terrestrial = absence of permanent water, o aquatic = presence of water, ◊ phycobiontic or endophytic, and □ unknown) and saline (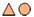); nonsaline (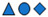); other extremes (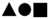 e.g., acidity); and unknown (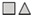) habitats. ^1^ Identical to *Watanabea* sp. NN-1-2-X1B (MT968415) and ^2^ identical to *Pseudostichococcus monallantoides* SY-1-2-P (MT991549), WT-3-1-A (MW074294), WT-3-1-H (MT991551), and WT-3-1-P1 (MT991552).

**Figure 6:**
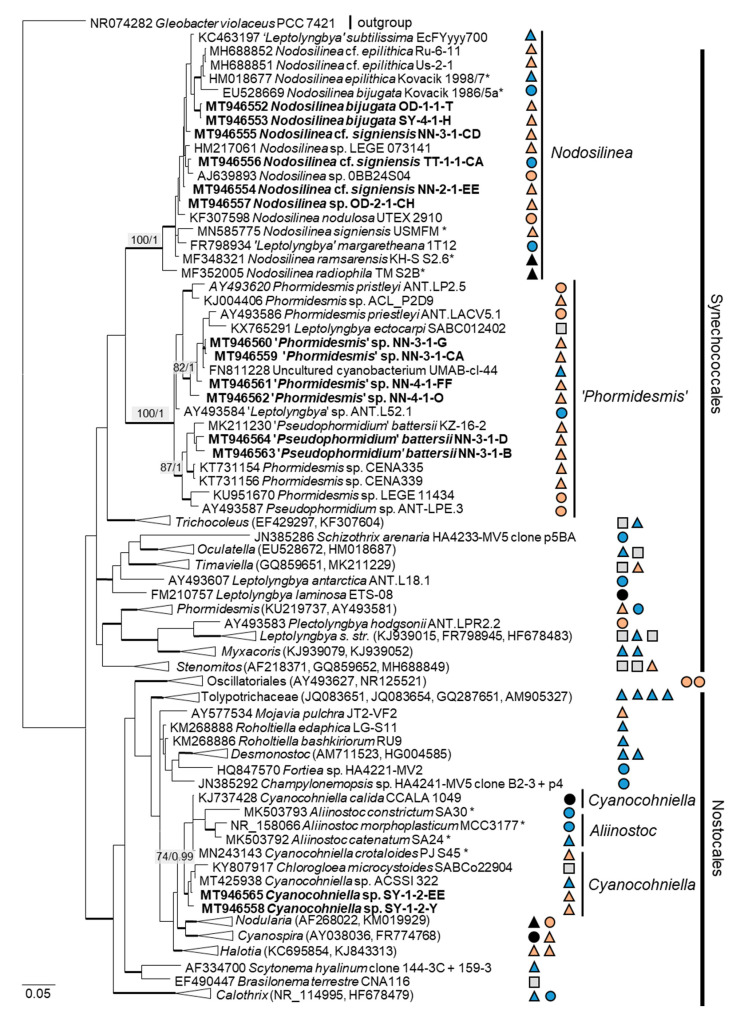
Molecular phylogeny of the cyanobacteria based on SSU rRNA gene sequencing. The phylogenetic tree was calculated by the Bayesian method, including posterior probabilities (PP) with additional maximum likelihood bootstrap values (BP); branches are supported by both methods in bold (BP > 60% and PP > 0.9). Authentic strains were marked with an asterisk, original strains from this study in bold. Habitats of the strains were classified according to habitat type (Δ terrestrial = absence of permanent water, o aquatic = presence of water, ◊ phycobiontic or endophytic, and □ unknown) and saline (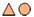); nonsaline (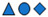); other extremes (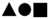 e.g., radioactivity); and unknown (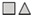) habitats.

Twenty-two percent (14 isolates) of the original strains belonging to Chlorophyta were distributed among the Ulvophyceae (Figure 4). They clustered in the order Ulvales, *Desmochloris*-clade (genera *Desmochloris* and *Halochlorococcum*), as well as in the order Ulotrichales, *Planophila*-clade (genus *Planophila*) and *Acrosiphonia*-clade (a separate lineage close to the clade with “*Ulothrix*” and *Chlorothrix* species). The majority of strains (45% of the Chlorophyta strains) fell in the Trebouxiophyceae (28 isolates, Figure 5) and were distributed among the genera *Chloroidium*, *Watanabea*, *Diplosphaera*, *Pseudostichococcus, Pseudochlorella*, *Chlorella*, and *Nannochloris*. Fourteen percent (two isolates) of the cyanobacteria isolates (Figure 6) clustered in the Nostocales (genus *Cyanocohniella*), and 86% (12 isolates) fell in the Synechococcales (genus *Nodosilinea* and an unresolved clade labeled “*Phormidesmis*”).

### 3.2. ITS Phylogeny

**Figure 7:**
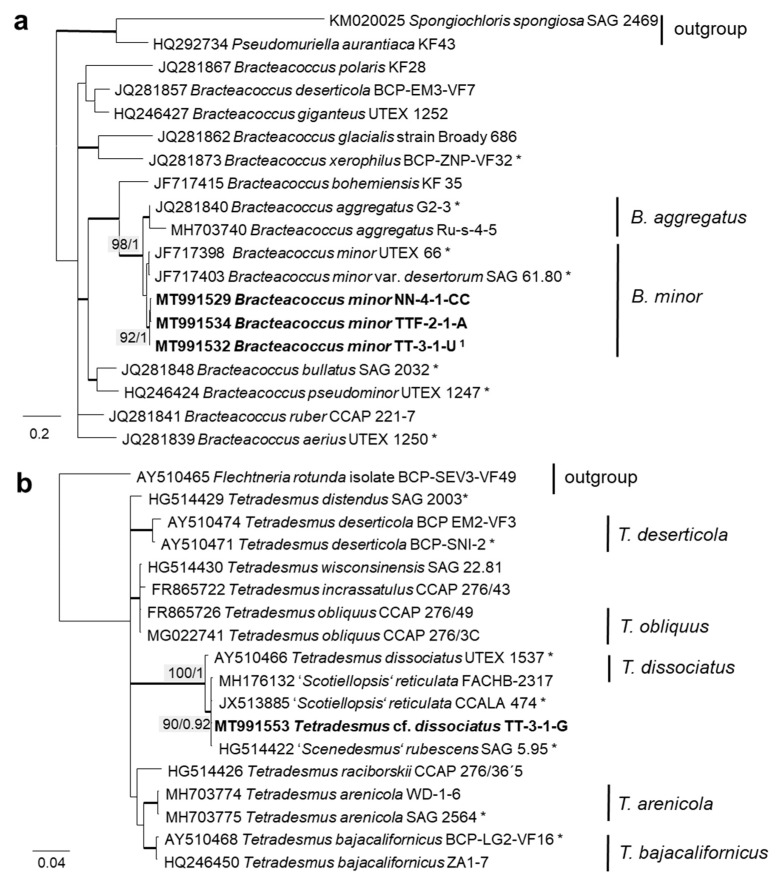
Molecular phylogeny of Sphaeropleales genera based on ITS-1,2 sequencing. (**a**) *Bracteacoccus* (only ITS-2) and (**b**) *Tetradesmus*. The phylogenetic tree was calculated by the Bayesian method, including posterior probabilities (PP) with additional maximum likelihood bootstrap values (BP); branches are supported by both methods in bold (BP > 60% and PP > 0.9). Authentic strains marked with an asterisk, original strains from this study in bold. ^1^ Identical to *Bracteacoccus minor* TTF-1-1-M (MT991533), TTF-2-1-J (MT991535), TT-3-1-J (MT991531), NN-4-1-D2 (MT968390), and NN-4-1-H (MT991530).

**Figure 8:**
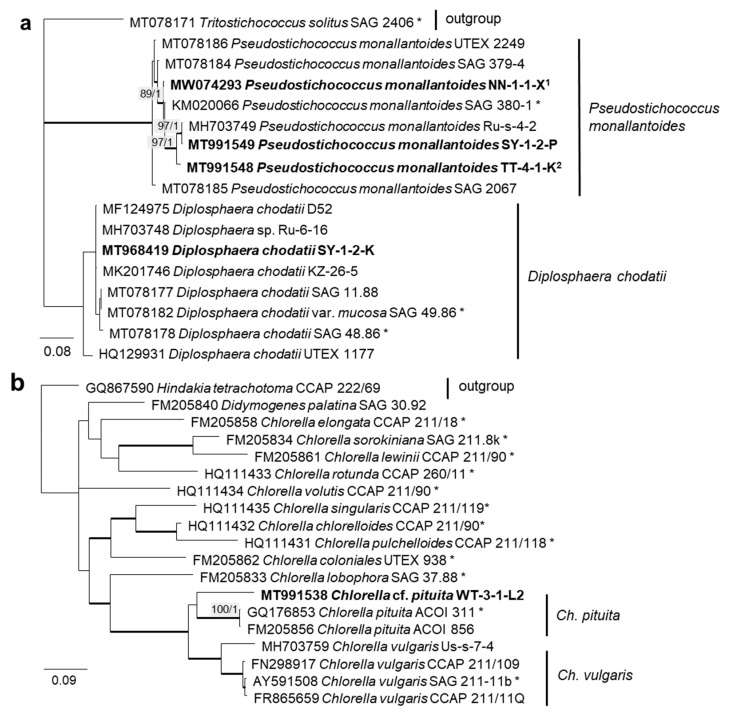
Molecular phylogeny of Trebouxiophyceae genera based on ITS-1,2 sequencing. (**a**) *Pseudostichococcus* and *Diplosphaera* and (**b**) *Chlorella*. The phylogenetic tree was calculated by the Bayesian method, including posterior probabilities (PP) with additional maximum likelihood bootstrap values (BP); branches are supported by both methods in bold (BP >60% and PP > 0.9). Authentic strains were marked with an asterisk, original strains from this study in bold. ^1^ Identical to *Pseudostichococcus monallantoides* WT-3-1-H (MT991551) and WT-3-1-P1 (MT991552). ^2^ Identical to *Pseudostichococcus monallantoides* TTF-2-1-BB (MT991550).

**Figure 9:**
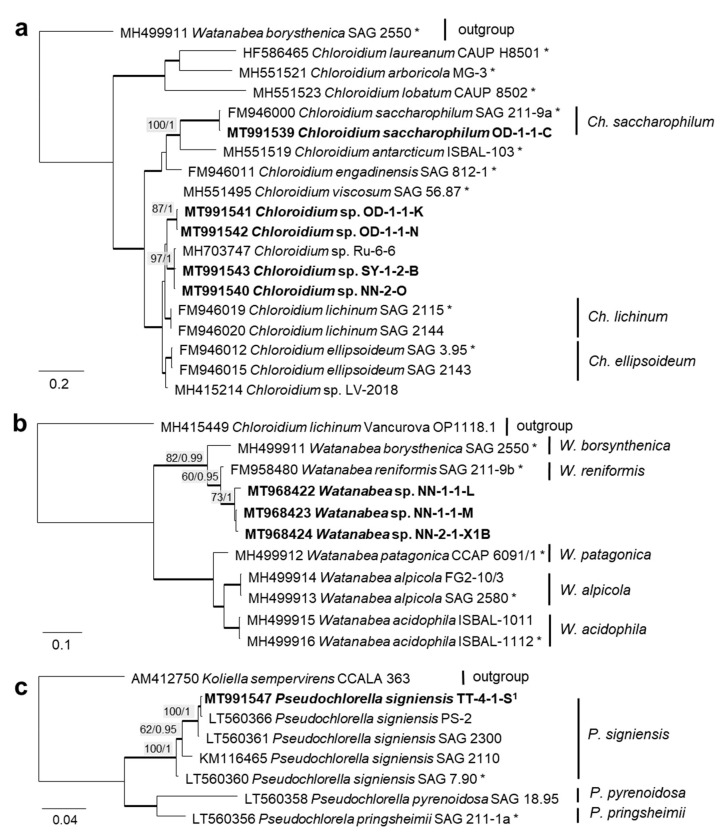
Molecular phylogeny of the Trebouxiophyceae genera based on ITS-1,2 sequencing (2). (**a**) *Chloroidium*, (**b**) *Watanabea*, and (**c**) *Pseudochlorella*. The phylogenetic tree was calculated by the Bayesian method, including posterior probabilities (PP) with additional maximum likelihood bootstrap values (BP); branches are supported by both methods in bold (BP > 60% and PP > 0.9). Authentic strains were marked with an asterisk, original strains from this study in bold. ^1^ Identical to *Pseudochlorella signiensis* TSN1 (MT968421) and TSN3 (MT991546).

In order to define the original strains more precisely (to the species level), ITS-1,2 phylogenies of several Chlorophyta and cyanobacteria genera were calculated. Some original strains of the Sphaeropleales, Chlorophyceae, and Trebouxiophyceae clearly clustered in clades formed by known species: *Bracteacoccus minor* (Figure 7a), *Pseudostichococcus monallantoides*, *Diplosphaera chodatii* (Figure 8a), *Chloroidium saccharophilum* (Figure 9a), and *Pseudochlorella signiensis* (Figure 9c). A Tetradesmus strain clustered within the strongly supported group formed by several species perhaps representing the same taxon (“*Scenedesmus” rubescens*, “*Scotiellopsis” reticulata*, and *Tetradesmus dissociatus* (Figure 7b).

**Figure 10:**
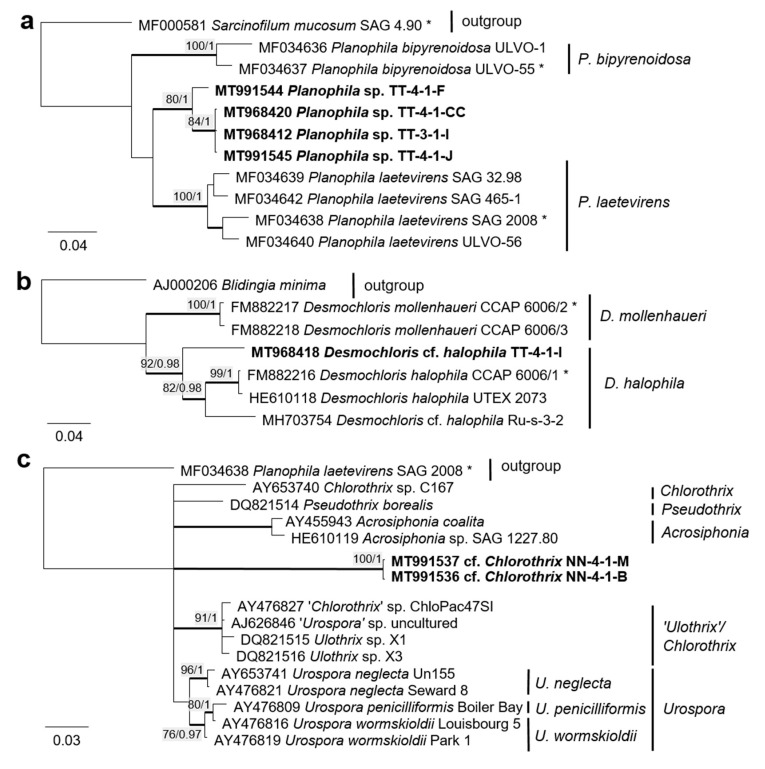
Molecular phylogeny of selected genera of the Ulvophyceae based on ITS-1,2 sequencing. (**a**) *Planophila*, (**b**) *Desmochloris*, and (**c**) *Acrosiphonia*-clade. The phylogenetic tree was calculated by the Bayesian method, including posterior probabilities (PP) with additional maximum likelihood bootstrap values (BP); branches are supported by both methods in bold (BP > 60% and PP > 0.9). Authentic strains marked with an asterisk, original strains from this study in bold.

Some original strains clustered with strains of known species but formed distant sister lineages to the authentic strains possibly representing other taxa: *Chlorella* cf. *pituita* (Figure 8b) and *Desmochloris* cf. *halophila* (Figure 10b). Many other original strains were placed in separate lineages from the reference strains of known species and may represent still unknown species. This was the case in the Trebouxiophyceae: *Chloroidium* (the closest species was *C. lichinum*, Figure 9a) and *Watanabea* (closest species *W. reniformis*, Figure 9b), as well as the Ulvophyceae: *Planophila* (the closest species was *P. laetevirens*, Figure 10a). In the *Acrosiphonia*-clade of the Ulvophyceae, two original strains (cf. *Chlorothrix*) formed a strongly distinct separate branch without closely related reference strains, which may represent a new genus (Figure 10c).

**Figure 11:**
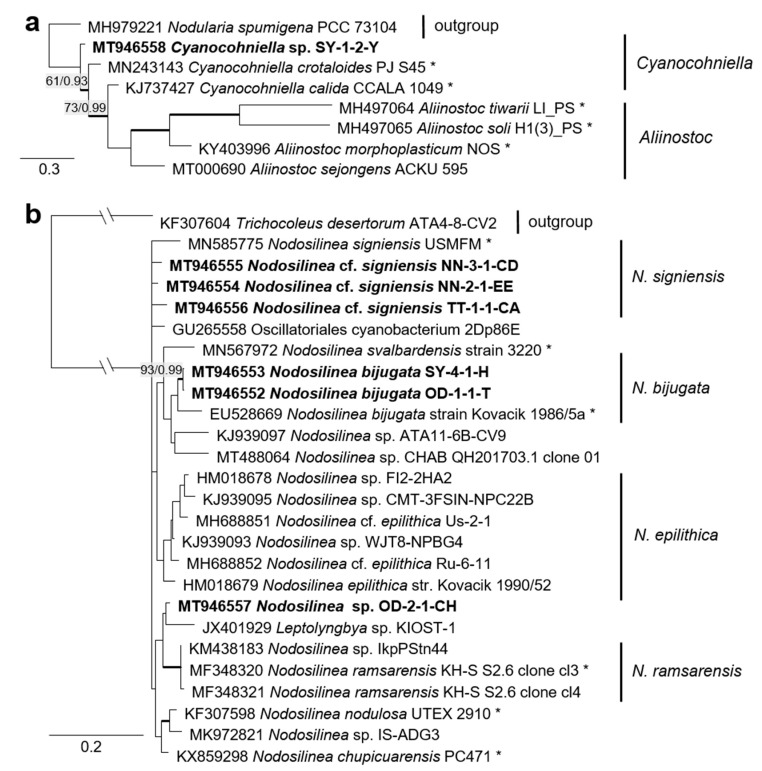
Molecular phylogeny of selected genera of the cyanobacteria. (**a**) *Cyanocohniella* based on SSU-LSU ITS sequencing and (**b**) *Nodosilinea* based on combined *SSU* and SSU-LSU ITS sequencing. The phylogenetic tree was calculated by the Bayesian method, including posterior probabilities (PP) with additional maximum likelihood bootstrap values (BP); branches are supported by both methods in bold (BP > 60% and PP > 0.9). Authentic strains marked with an asterisk, original strains of this study in bold.

The combined SSU rRNA and SSU-LSU ITS phylogeny of the genus *Nodosilinea* (Synechococcales, cyanobacteria, Figure 11b) showed that two original strains clearly clustered with the species *N. bijugata*; the other four strains formed lineages close to the species *Nodosilinea signiensis* and the unidentified strain *Nodosilinea* KIOST-1. The SSU-LSU ITS phylogeny of a Nostocales isolate showed closeness to the *Cyanocohniella* and *Aliinostoc* species, rendering a paraphyletic position of *Cyanocohniella* (Figure 11a).

### 3.3. Morphological Observations

**Figure 12:**
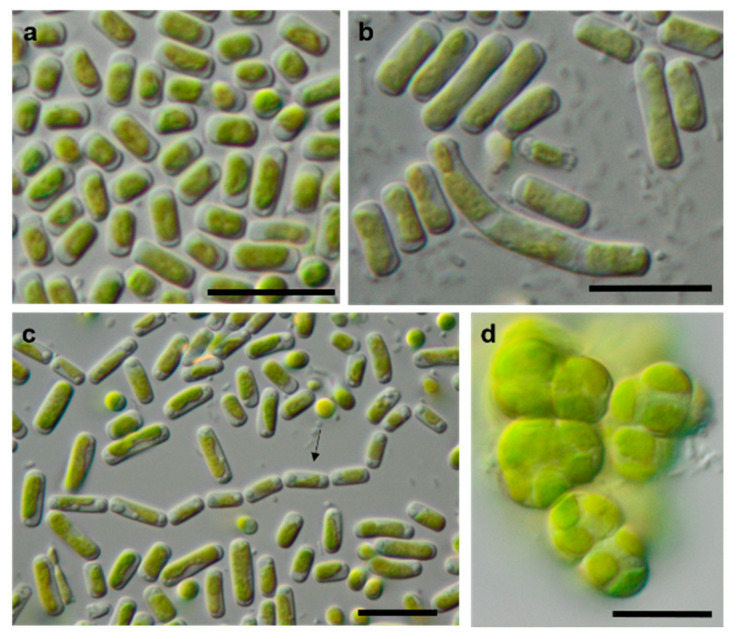
Photomicrographs of Trebouxiophyceae isolates. (**a**,**c**): *Pseudostichococcus monallantoides* WT-3-1-A (**a**) young cells and (**c**) mature cells forming a filament (arrow)), (**b**) *Pseudostichococcus monallantoides* SY-1-2-X, and (**d**) *Diplosphaera chodatii* SY-1-2-K with cubic cell packages. Scale bars: 10 µm.

**Figure 13:**
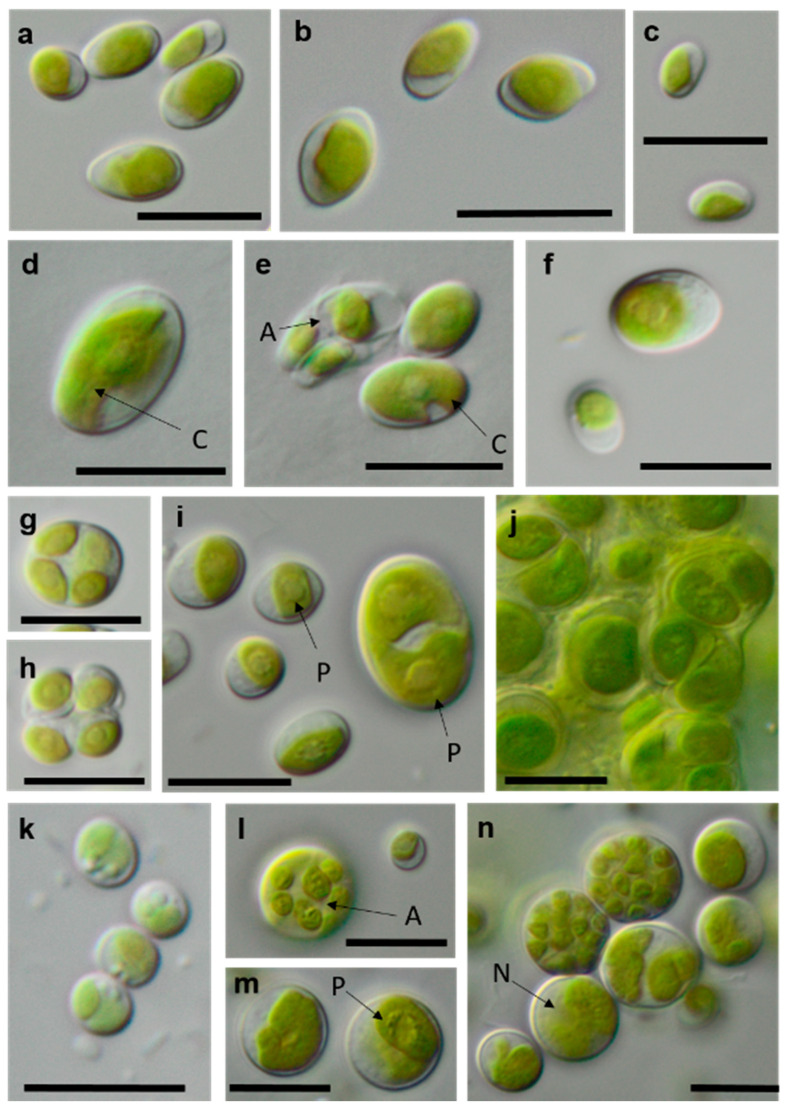
Photomicrographs of Trebouxiophyceae isolates (2). (**a**–**c**) *Chloroidium saccharophilum* OD-1-1-C with a smooth chloroplast; (**d**,**e**) *Chloroidium* sp. OD-1-1-K with lobed chloroplast (**d**) mature cell, (**e**) autospore, and mature cells; (**f**–**j**) *Pseudochlorella signiensis* TSN-1 (**f**) young cells, (**g**) autosporangium, (**h**) autospores, and (**i**) cells with sharp pyrenoids; (**j**) mature cells in cell aggregate; (**k**) *Nannochloris* sp. TT-3-1-F; and (**l**–**n**) *Watanabea* sp. NN-1-1-E (**l**) autosporangium, (**m**) mature cells with parietal chloroplast, and prominent pyrenoid. Arrow labels: A = autospore, N = nucleus, and P = pyrenoid. Scale bars: 10 µm.

**Figure 14:**
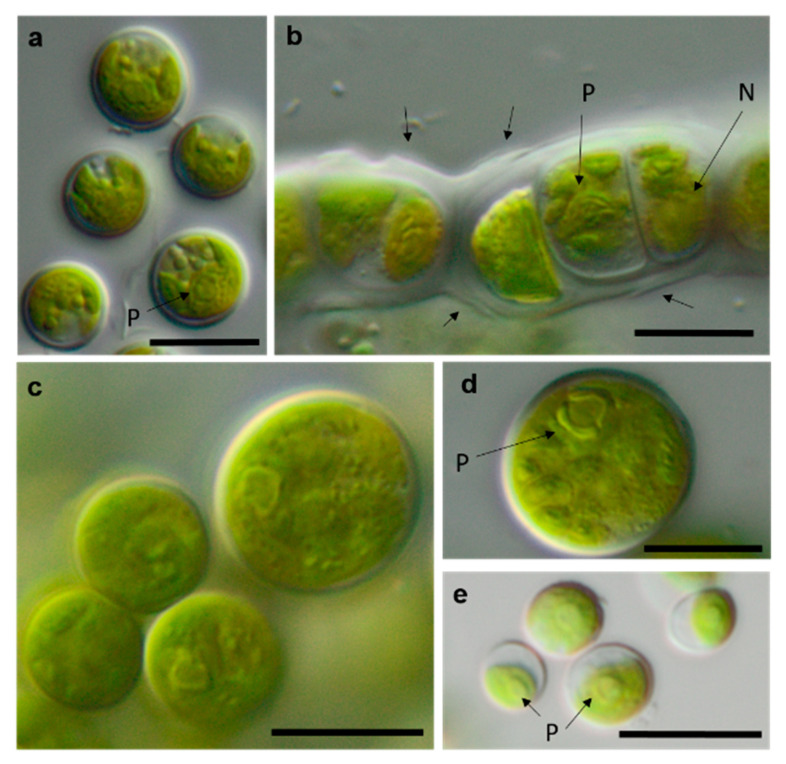
Photomicrographs of Ulvophyceae isolates. (**a**) *Desmochloris* cf. *halophila* TT-4-1-I, (**b**) cf. *Chlorothrix* NN-1-1-M a corrugated cell wall, (**c**,**d**) *Halochlorococcum* sp. TT-4-1-O (**d**) single cell with lateral pyrenoid surrounded by two starch grains, and (**e**): *Planophila* sp. G2C with an unlobed chloroplast and one pyrenoid. Arrow labels: N = nucleus and P = pyrenoid. Scale bars: 10 µm.

**Figure 15:**
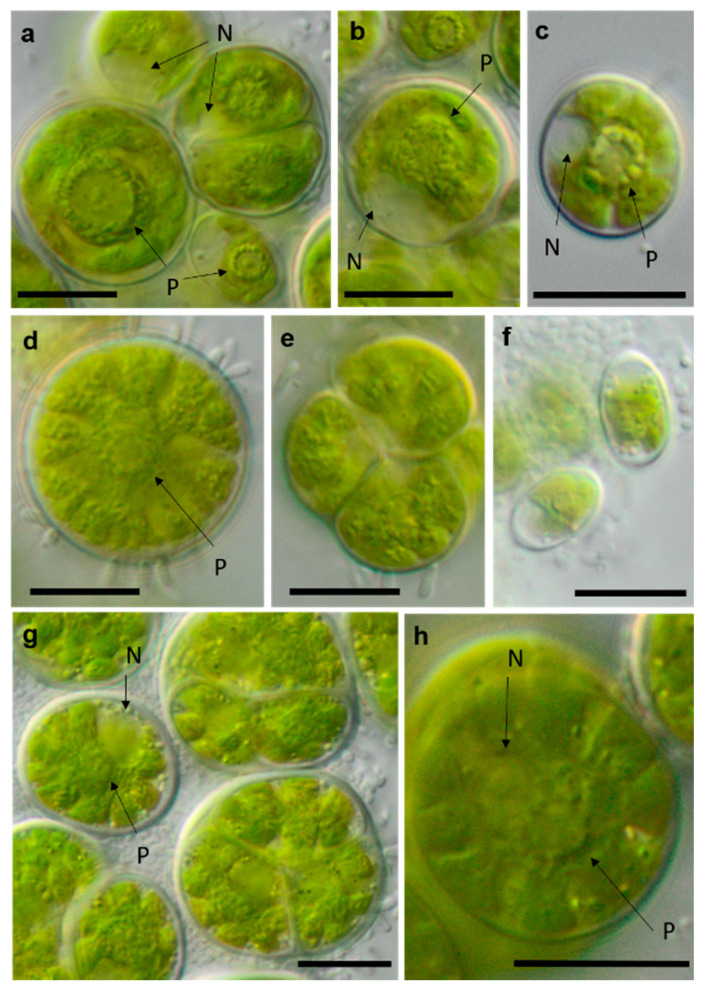
Photomicrographs of Chlamydomonadales isolates. (**a**,**b**) cf. *Spongiococcum* TT-3-1-M; (**c**–**e**) *Borodinellopsis* sp. NN-2-D (**c**) juvenile cell and (**d**) mature cell with lobed chloroplast; and (**f**–**h**) *Borodinellopsis texensis* NN-4-1-E (**f**) zoospores, (**h**) single cell with prominent nucleus, and one pyrenoid surrounded by several fine starch grains. Arrow labels: N = nucleus and P = pyrenoid. Scale bars: 10 µm.

**Figure 16:**
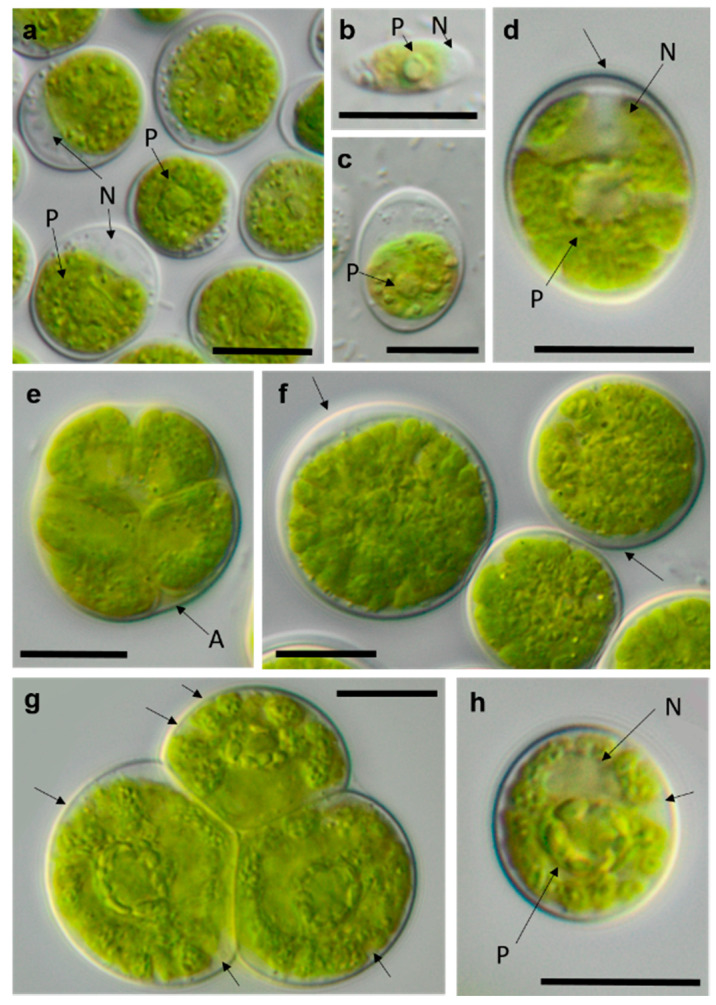
Photomicrographs of Chlamydomonadales isolates (2). (**a**–**c**) cf. *Chlorogonium* SY-1-2-T (**a**) mature cells and (**b**) zoospore; (**d**–**f**) *Chloromonas* sp. WT-3-1-F (**d**) young cell, (**e**) zoosporangium, and (**f**) mature cell with apical cell wall bulge (arrow); and (**g**,**h**) cf. *Axilosphaera* NN-1-1-Q, arrows indicating slightly open spaces between chloroplast lobes ((**g**) adult cells in tetrad and (**h**) young cell. Arrow labels: N = nucleus and P = pyrenoid. Scale bars: 10 µm.

**Figure 17:**
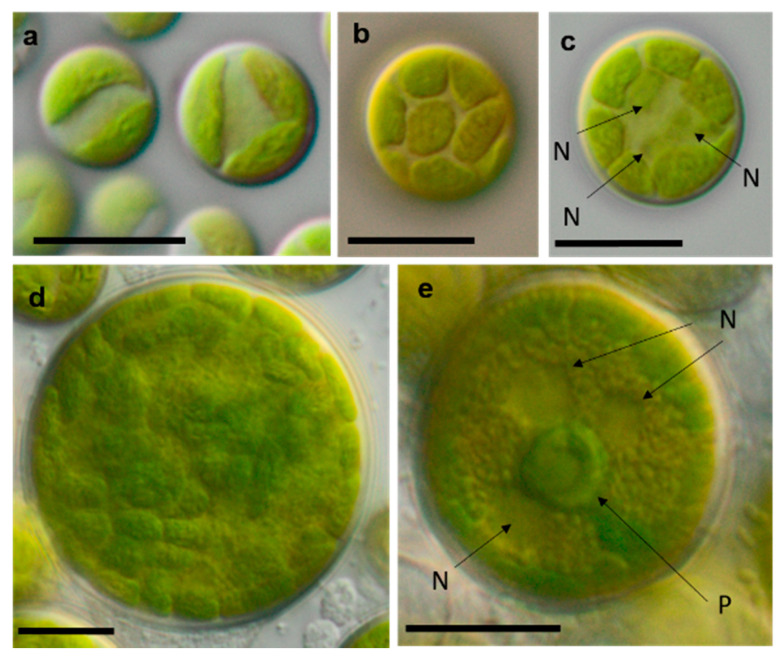
Photomicrographs of Sphaeropleales isolates. (**a**–**d**) *Bracteacoccus minor* (**a**) young cells, TTF-2-1-M and (**b**,**c**) TTF-2-1-A (**b**) surface view of disc-shaped chloroplasts; (**c**) several nuclei are visible in the cell; (**d**) mature cell with multiple chloroplasts, NN-4-1-CC, (**e**) and *Tetradesmus* cf. *dissocuatus* TT-3-1-G, a large mature cell before division containing several nuclei and a central pyrenoid. Arrow labels: N = nucleus and P = pyrenoid. Scale bars: 10 µm.

**Figure 18:**
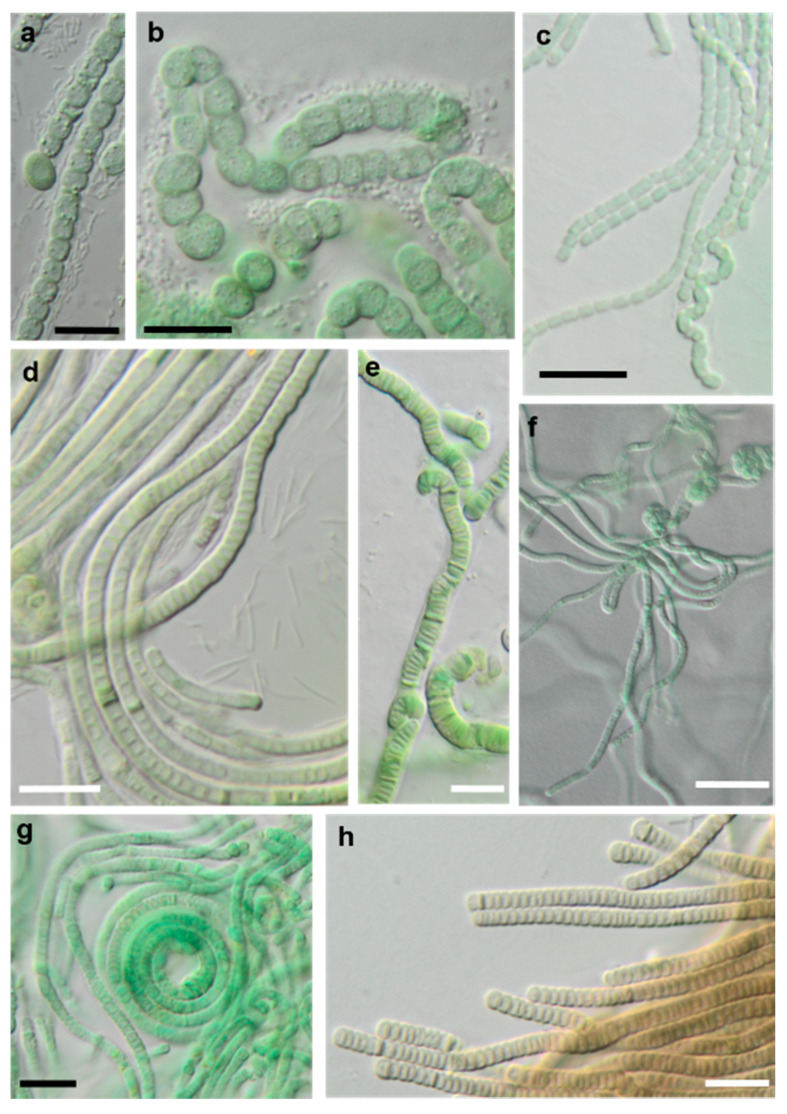
Photomicrographs of cyanobacteria isolates: (**a**,**b**) Nostocales and (**c**–**h**) Synecchococcales. (**a**,**b**) *Cyanocohniella* sp. SY-1-2-Y, (**c**) *Nodosilinea* cf. *signiensis* NN-3-1-CD, (**d**) “P*hormidesmis*” sp. NN-3-1-CA, (**e**) “*Pseudophormidium*” *battersii* NN-3-1-D, (**f**) *Nodosilinea* cf. *signiensis* NN-2-1-EE, (**g**) *Nodosilinea* *bijugata* OD-1-1-T, and (**h**) “*Pseudophormidium*” *battersii* NN-3-1-B. Black scale bars: 10 µm and white scale bars: 20 µm.

The morphology of the original strains was examined and is presented on Figure 12, Figure 13, Figure 14, Figure 15, Figure 16, Figure 17 and Figure 18. Forty strains were unambiguously identified to the species level using morphological characters solely. Five strains were tentatively identified because of unclear morphological characters or differences from morphological diagnoses of some species. Thirty-one strains were identified only to the genus level or higher because of morphological inconsistencies with known species or the absence of clear morphological characters. The list of these morphological species is included in Table 1 and has been published previously [63].

## 4. Discussion

### 4.1. Morphological vs. Molecular Species Determination

Table 1 shows a comparison of the results from our study to the previously published morphological species list [63]. Several of the original isolates were previously correctly identified to the species level based solely on morphology. Usually, these taxa have distinct morphological features that can be recognized quickly. As an example, *Borodinellopsis texensis* is characterized by a prominent asteroid chloroplast, central pyrenoid, and the formation of cell packages (Figure 15f–h), and *Diplosphaera chodatii* is identified by the characteristic cubic cell packages, cell diameter, and the absence of coated pyrenoids (Figure 12d) [100].

Some strains were correctly identified to the genus level or morphological species complexes. For example, several strains of the morphologically prominent genus *Chloroidium,* initially identified as *Ch. ellipsoideum*, were redefined as the morphologically close species *Ch. saccharophilum* and *Chloroidium* sp. The cells of this genus can be easily recognized by their ellipsoid shapes and characteristic chloroplast structures (Figure 13a–e) [68]. Previous identifications of the small-celled *Nannochloris* sp. were also confirmed by the integrative approach. However, the group of *Nannochloris*-like algae is problematic for identification in general and requires taxonomic revision, including strains from ecologically different environments [101,102].

Several strains with *Tetracystis*/*Chlorococcum*-like morphology finally fell in different but related lineages in the Moewusinia (Chlorophyceae; *Axilosphaera*, *Spongiococcum*, *Alvikia*, and *Chlorogonium*), confirming the general polyphyly of this morphotype [103]. The strain that was morphologically identified as *Radiosphaera negevensis* (Moewusinia) due to the presence of almost spherical mature cells and typical asteroid chloroplasts was redefined as the *Chloromonas* sp. (Chloromonadinia). This disagreement in identification may occur due to the high level of morphological parallelism between nonrelated lineages of Chlorophyceae, previously discussed by Mikhailyuk et al. [65]. Possibly, the same explanation can be applied to the strains cf. *Chlorothrix* (Acrosiphonia-clade, Ulotrichales, and Ulvophyceae), which were originally identified as *Ulothrix* species (Figure 4, *Planophila*-clade).

Further, the identification of some cryptic taxa characterized by simple morphology was corrected based on the integrative approach. Several strains that were initially identified as morphologically different *Stichococcus* species were unambiguously identified as a single species in the newly revised genus *Pseudostichococcus* [119] (Figure 8a), namely *P. monallantoides.* Likewise, some strains of *Bracteacoccus minor* were initially morphologically identified as several small-celled *Bracteacoccus* (including *B. minor*) and *Pseudomuriella* species (Figure 7a).

The *Parietochloris* strains that were morphologically identified due to their specific structure of the pyrenoid envelope, chloroplast morphology, and general appearance of young and mature cells [100], however, were redefined as the recently revised genera *Pseudochlorella* and *Watanabea* [70,121]. Members of the same class (Trebouxiophyceae), these genera are related and, consequently, have similar morphology.

The strains morphologically identified as *Spongiochloris excentrica* (Chlorophyceae) mostly clustered in a completely different class (Ulvophyceae) in clades corresponding to the genera *Desmochloris* and *Halochlorococcum*. This may have occurred due to morphological similarities between the two classes: a high similarity of the spongiomorph chloroplast and the pyrenoid morphology, as well as the multinucleosis of vegetative cells in some stages of their life cycles. These morphological parallelisms have been discussed in a recent publication [65]. *Halochlorococcum* is poorly investigated [114] and absent from widely used identification books and guides [100], which underlines the difficulty of identifying this genus morphologically.

The strain TT-3-1-G was also identified as *Spongiochloris excentrica* on the basis of morphological features [100]; however, following the phylogenetic analysis, it clustered in another chlorophycean genus. This genus, *Tetradesmus*, is morphologically completely different from *Spongiochloris*. Our strain formed large multinucleate cells (see Figure 17e) that actually resemble *Spongiochloris*. These cells may represent a specific stage in the life cycle of *Tetradesmus* (before division and autospore formation) rather than a general phenotype.

The strains morphologically identified as *Nostoc* sensu lato and *Leptolyngbya* sensu lato, to give an example for cyanobacteria, were redetermined to be species of the newly described genera *Cyanocohniella* [76] and *Nodosilinea* [122], which still belong to the same orders (Nostocales and Synechococcales, respectively). Altogether, the molecular approach resulted in more precise identification and should be considered as an extension of the previous morphological species list.

As explained above, some of these misidentifications resulted in the splitting of one taxon into several morphologically identified taxa. This can be explained by two factors: age of cultures and phenotypic plasticity. Cultures of different ages may have shown differences in cell morphology. Young cells may be smaller and, for example, in the case of *Halochlorococcum* sp., are more likely to resemble *Chlorella*. Older cells may have been larger and potentially showed more morphological traits that were attributed to another taxon. Likewise, young cells of *Planophila* sp. did not form characteristic cell packets but showed a *Chlorella*-like morphology (Figure 14e), leading to the mismatch between the morphological approach and the molecular results.

Differences in morphology also appear with different culture conditions, such as temperature, light, and salinity. This effect is termed phenotypic plasticity. Some recent studies have revealed that phenotypic plasticity of several terrestrial green algae can be caused by different levels of salinity [119,124].

Some morphologically identified species from the potash tailing pile sites [63] could not be addressed with the methods used here. One example is the genus *Pseudendoclonium*, a filamentous branched green alga in the Ulvophyceae, which was frequently found in biocrusts of the tailing pile environments [63]. This genus is a widely distributed halotolerant alga and has also been identified as *Dilabifilum* in some studies [43,51,125]. In the previous study, the genus *Planophila* was represented by five morphologically identified species [63], e.g., characterized by dense cell aggregates. However, the present study identified only one species, which was single-celled (Figure 14e). Sequencing other morphotypes of this genus was not successful. Thus, our approach was not feasible for these isolates. For these reasons, the taxa identified in this study do not comprise a complete species list.

In sum, the integrative approach confirmed some of the morphological taxa, redefined others more precisely, and excluded a few strains due to methodological issues. Consequently, the results should be considered as an extension and revision of the species list published previously [63], underlining the general advantages of an integrative approach.

### 4.2. Taxa with Unclear Phylogenetic Positions

In the Chlamydomonadales, the genus *Borodinellopsis* was isolated quite often from our samples (four isolates, Figure 15c–h). Two original strains were clearly identified as *Borodinellopsis texensis*. Another two original strains formed a sister branch to this species (Figure 2). This lineage may represent another species of *Borodinellopsis*, either *B. oleifera*, which is morphologically characterized by the presence of oil drops [126], or a new taxon. However, no reference sequence of *B. oleifera* was available. Thus, more detailed morphological observation is necessary to reach a definitive conclusion.

Several strains were found among the *Tetracystis*/*Chlorococcum*-like isolates in the Moewusinia-clade, identified as cf. *Axilosphaera*, cf. *Spongiococcus*, and *Alvikia* sp. The isolate NN-1-1-Q (cf. *Axilosphaera*, Figure 16g,h) clustered with *Eubrownia* and *Axilosphaera vegetata*. Even though the latter represents the closest relative, the isolate analyzed in this study differed from these reference strains regarding the SSU rRNA gene sequence, indicating a possibly new taxon. Ettl and Gärtner [100] described *Axilosphaera vegetata* as morphologically similar to *Borodinellopsis* but distinguished by the absence of the larger cell complexes in *Axilosphaera*, which, in turn, are common for *Borodinellopsis*. We observed the isolate NN-1-1-Q in dyads or tetrads only (Figure 16g), which showed parietal cup-shaped and richly dissected chloroplasts (Figure 16g,h). The chloroplast of *Axilosphaera* is described as parietal cup-shaped and dissected, becoming almost asteroid in mature cells, which is similar to the cup-shaped and dissected chloroplasts of the sister lineage *Eubrownia* [105]. Two other Moewusinia isolates fell in new lineages (Figure 2). The isolates cf. *Spongiococcum* (SY-4-1-C and TT-3-1-M), which showed a *Tetracystis*-like morphology and a spongiomorph chloroplast with a prominent pyrenoid (Figure 15a,b), as well as the isolate *Alvikia* sp. (TTF-2-1-Da), which showed a *Chlorococcum*-like morphology, possibly representing new taxa. For final clarification, more thorough investigations are required.

The original isolates TT-3-1-Q, SY-1-2-T and TSN3f, placed in the Chlorogonia, were unique, since they were clearly separated from the closest *Chlorogonium* species. The genus *Chlorogonium* is flagellated, spindle-shaped, or ovoid and characterized by the presence of two or more pyrenoids [103]. Young cells of our isolates had *Chlorogonium*-like morphology (Figure 16b) and had one pyrenoid and a posterior nucleus; however, mature cells became ovoid to nearly spherical, never a spindle shape (Figure 16a,c). Consequently, our isolates may represent a new taxon.

Several strains belonged to the Ulvophyceae. In the order Ulvales, the isolate T-4-I-1 fell in the genus *Desmochloris* (Figure 4 and Figure 14a), which includes two species: *D. mollenhauerii* and *D. halophila* [112]. The original strain of this study was clearly separated from *D. mollenhauerii* and was placed in an intermediate position to the type of species of *D. halophila.* Thus, we identified our strains as *Desmochloris* cf. *halophila*, which may represent a new species for the genus.

Several other isolates within the *Desmochloris*-clade were placed in the interesting and insufficiently described genus *Halochlorococcum* (according to the SSU rRNA gene phylogeny, Figure 4). Seven species are known in this genus [114]. Even though the genus was recently re-established after having an invalid status for a long time [114], only the SSU rRNA gene sequences of three species (*H. dilatatum*, *H. porphyrae*, and *H. moorei*) were available in the database, which did not allow specification of the original isolates to the species level. This stresses the need for a general revision of the genus *Halochlorococcum*.

Four original strains of another ulvophyte fell in the genus *Planophila*, forming a distinct separate lineage between the two known species *P. bipyrenoidosa* and *P. laetevirens* (according to the ITS-1,2 phylogeny, Figure 10a). This result strongly indicates that these strains represent a new species.

A unique case consists of two original strains from the Ulotrichales. According to the ITS-1,2 phylogeny, these strains were placed in the *Acrosiphonia*-clade (Figure 4), forming a significant new branch close to the genera *Pseudothrix*, *Chlorothrix*, *Urospora*, and “*Ulothrix*” (Figure 10c). *Urospora* is a filamentous, uniseriate marine algae and has a multinucleate gametophyte [116], whereas the gametophyte thallus of *Pseudothrix* is initially uniseriate and develops into multiseriate filaments [117]. Mature cells of *Ulothrix* and *Chlorothrix* are uniseriate and uninucleate [127]. Thus, our isolates showing uniseriate and uninucleate filaments (Figure 14b) were morphologically most similar to *Ulothrix* and *Clorothrix*. The genus *Ulothrix*, however, is placed in the *Planophila*-clade of the Ulotrichales [69] (Figure 4), represented by strains identified as *Ulothrix zonata* (SAG 38.86, UTEX 745). Thus, we tentatively named our isolates (NN-4-1-M and NN-4-1-B) cf. *Chlorothrix*.

Several different cultures were found in the Trebouxiophyceae. Five original isolates clustered within the genus *Chloroidium* (Figure 5). One of them (OD-1-1-C) was clearly identified as *Ch. saccharophilum*, since its ITS-1,2 sequence was identical to the authentic strain of this species (SAG 211-9a, Figure 9a). The other isolates formed two separate lineages together with the unidentified strain, originating from sand dunes of the Baltic Sea coast (Ru-6-6). These lineages are close to *Ch.lichinum*, as well as to *Ch. ellipsoideum*, and may represent new species.

Three other trebouxiophycean isolates clustered with *Watanabea* and formed a separate lineage closely related to the type strain of *W. reniformis* (SAG 211-9b). The chloroplast of *W. reniformis* does not show a pyrenoid [70], whereas the isolates of this study clearly did (Figure 13m). Another species in this clade, *W. borsynthenica*, in turn, has a pyrenoid [70]. However, the latter is clearly different from our isolates regarding the ITS-1,2 phylogeny, which leads to an unclear status of the original isolates.

In the Sphaeropleales, the original strain TT-3-1-G clustered in the genus *Tetradesmus*. It was close to the authentic strains of four species: “*Scenedesmus*” *rubescens*, “*Scotiellopsis*” *reticulata*, *Tetradesmus dissociatus*, and “*Scenedesmus*” *littoralis*, forming a highly supported cluster in the SSU rRNA gene and ITS-1,2 phylogenies (Figure 3 and Figure 7b). Indeed, the SSU rRNA gene and the ITS sequences were very similar (differing in only one nucleotide) and, also, in their ITS-2 region, which is used for species delimitation within the genus [128]. Thus, it is obvious that this cluster, including our original strain, most likely represents one distinct species. The findings of another study confirm the genetic similarity here [111]. However, there are some nomenclatural uncertainties, since the status of the oldest species name in that clade (*Halochlorella rubescens*) was recently declared as invalid [129]. Despite the genetic identity and a generally similar morphology of these four strains, they showed some minor, though characteristic, morphological differences: *Scenedesmus rubescens* forms cell packets [130], and *Tetradesmus dissociatus* shows characteristic thread-like remnants of the sporangial walls. We also observed some specific multinucleate stages in our original strain TT-3-1-G (Figure 17e), which may have caused its previous misidentification (see above). Therefore, further investigations are required for the final decision concerning this clade. Accordingly, we named the original isolate as *Tetradesmus dissociatus*, since it is the only valid species name available in this group.

The two cyanobacteria isolates in the Nostocales clustered with the rare and recently described and revised genus *Cyanocohniella* [75,76]. However, both the SSU and SSU-LSU intergenic spacer phylogeny showed a paraphyly of *Cyanocohniella* and the genetically close genus *Aliinostoc* (Figure 6 and Figure 11a). Therefore, the identification remains unclear. Moreover, both genera may require further revision.

Several original isolates from the Synechococcales with unclear *Leptolyngbya*-like morphology (Figure 18c,f,g) clustered within the genus *Nodosilinea*, which was defined recently [122], followed by the description of several new *Nodosilinea* species [123,131,132]. Some of our isolates fell in clades formed by known species (Figure 11b) and identified as *N. bijugata* and *N.* cf. *signiensis*. The original isolate OD-2-1-H, however, was placed in a distinct clade with an unidentified reference strain (KIOST-1), indicating its unclear status.

Six other original isolates belonging to Synechococcales and characterized by prominent, deeply constricted green, pale-green, and brown trichomes, as well as narrow cells (Figure 18d–h), were ordered into another clade containing sequences labeled as *Phormidesmis* and *Pseudophormidium*. However, a recent review addressed the genus *Phormidesmis* [133], which clearly represents another lineage. Previous studies that found strains of the same clade termed it “*Phormidesmis*” [65,72], which stresses its unclear status and, as discussed above, may represent an undescribed genus [72]. Clearly, identification of our strains is not possible.

Overall, our study showed a high proportion of unclear and possibly new taxa. For 24% of the taxa found in this study (16 isolates), identification even to genus level was doubtful or not possible from the integrative approach. This is twice as high as in a similar study on biocrusts in Baltic Sea coastal dunes, where the authors found unclear genera in 11% of their isolates [65]. Another recent study on microbial mats in hypersaline wind-tidal flats found that even 35% of the isolated cyanobacteria could not be assigned to the genus/family level [35]. This underlines the lack of knowledge in hypersaline environments, which is in congruence with our findings on biocrusts in the highly saline potash tailing pile environments.

The methods used in our study were not sufficient to formally describe the new taxa. Therefore, deeper analyses are needed. One crucial molecular method is the comparison of ITS secondary structures that clearly delimit the borders between species of both green algae and cyanobacteria. In addition, the morphology should be analyzed in detail. The original strains of this study were cultured on a solidified saline medium, which could result in changes in morphology. Phenotypic plasticity under saline conditions is known for several species [134]. To overcome this possible limitation, several growth media with or without added salt should be used, both solidified and liquid. In addition, the morphology of all reproductive stages, such as zoospores, gametes, or resting stages in green algae, as well as hormogonia or necridia in cyanobacteria, should be investigated in detail.

### 4.3. Congruence and Divergence of Phylogeny and Habitat Characteristics Regarding Salinity

Several species or genera identified in this study are already known to live in salt-affected habitats and, thus, are most probably adapted to saline conditions. It is not surprising that we identified many strains of Ulvophyceae, since this class mostly contains salt-tolerant marine species, such as the eponymous macrophyte genus *Ulva*. However, in terrestrial habitats, members of this class are mostly less common than the predominant Chlorophyceae and Trebouxiophyceae. Only a few studies have treated nonmarine Ulvophyceae, which were recently rearranged in the broad revision of Darienko and Pröschold [69].

The Ulvophyceae *Desmochloris halophila* was originally found in mixohaline water [113]. Other strains assigned to that genus/species also originated from salt-affected habitats [65,135]. Another Ulvophyceae, a close relative to the original strains of *Halochlorococcum* sp., was *Halochlorococcum dilatatum* (SAG 12.90), which was originally isolated from a rock pool in Helgoland, Germany. Rock pools have fluctuating salinities, which may strongly increase due to evaporation and reach hypersaline concentrations. The two other reference strains, *H. porphyreae* and *H. moorei*, were also found in marine environments [115,117]. Mettlig [136] classified the group as a genus, including soil algae; however, no record from a terrestrial environment was published thereafter. Thus, the findings of our study indicate the first record of *Halochlorococcum* in a biocrust.

The clade *Tetradesmus*, including “*Scotiellopsis*” *reticulata*, “*Scenedesmus*” *rubescens*, “*S.*” *littoralis*, and *T. dissociates*, is an interesting case in the Sphaeropleales concerning salinity. The reference strain “*Scotiellopsis*” *reticulata* (CCALA 474) was found in the sand on the coast of the Black Sea [137], and the type strain of “*Scenedesmus*” *littoralis* originated from coastal waters and tolerates up to 2% NaCl [138]. “*Scenedesmus*” *rubescens* was described based on a strain isolated from a culture of marine brown algae [130]. The reference strain *Tetradesmus dissociatus* is the only exception, since it was isolated from cornfield soil [139].

The order Chlamydomonadales also contains salt-tolerant species such as *Borodinellopsis texensis*, which was found in a sea salt farm (strain JM002, Figure 2) and in a hypersaline ecosystem [43]. The isolate *Alvikia* sp. (TTF-2-1-Da), interestingly, clustered with species found in saline environments [105] but not in terrestrial habitats. In contrast, the sister clade with *Spongiococcum* and *Actinochloris* included terrestrial isolates from nonsaline environments. Thus, our isolate may somehow represent a linkage between these clades as the first record from a salt-affected terrestrial site.

In the cyanobacteria, the closest reference to the two original strains, *Cyanocohniella* sp., was originally isolated from beach mats of the North Sea, The Netherlands [75]. In the sister genus *Aliinostoc*, some strains are known from other saline habitats, such as saline-alkaline lakes or mangroves [140]. In the “*Phormidesmis*” cluster, almost all of the respective reference strains were originally isolated from salt-affected habitats but of different types. These references originated from microbial mats in saline lakes [141,142] and from the coast of the brackish Sea of Azov [72]. Two strains of “*Pseudophormidium*” were isolated from the unusual habitat of salt-excreting leaves of the mangrove *Avicennia schaueriana* [143]. In sum, the original isolates belonging to the taxa discussed above match the saline habitat preferences of their closest phylogenetic relatives.

In contrast, several genera were identified that are more common in nonsaline environments. The Trebouxiophyceae comprises a wide variety of microalgae, including marine and freshwater, as well as numerous terrestrial taxa. In the genus *Watanabea*, most species were described from either aquatic, e.g., *Watanabea reniformis* (SAG 211-9b), or terrestrial habitats; there is no record from saline terrestrial environments yet known. Interestingly, several strains have been isolated from highly acidic environments [70,144]. Thus, the original isolates of this study underline the presence of extremophiles within the genus *Watanabea* and represent a unique finding in a saline biocrust.

A study focusing on *Nannochloris*-like algae described a new genus of marine/saline *Nannochloris*-like strains, namely *Picochlorum*, whereas freshwater *Nannochloris*-like strains formed a second cluster [101]. The respective strains of our study did not fall into the marine *Picochlorum* but, rather, in the freshwater *Nannochloris*.

The Trebouxiophyceae genus *Chlorella* is among one of the best-known genera of microalgae, but its phylogenetic status has long remained unresolved. A recent study addressed the question of the existence of true marine *Chlorella* species and, indeed, found three marine isolates belonging to this genus, namely *Chlorella vulgaris* [145]. The original isolate of this study, however, clustered with *Chlorella pituita*. The epitype strain of *Chlorella pituita*, though, was isolated from freshwater [97].

Consequently, we found several isolates that represent taxa that are typically found in saline habitats or that have some close relatives that inhabit such environments. Other taxa were more typical for nonsaline habitats. To withstand the harmful saline environment, salt-tolerant microalgae evolved protective mechanisms such as the excretion of extracellular polysaccharides (EPS) and the production of intracellular organic osmolytes. However, the biocrust as a multidimensional layer potentially provides shelter for less salt-tolerant organisms. Species adapted to salty conditions may form a barrier layer for sensitive species by EPS excretion. Thus, both tolerant as well as nontolerant taxa may appear in salt-affected biocrusts, although intolerant taxa may not survive outside the shelter of the biocrust community. Further, we cannot state whether the species lived under their optimum conditions or at the edge of their salt tolerance. Competitive pressure in less saline soils in these environments may lead to a migration of taxa to more saline soils on the edge of their tolerance. Future eco-physiological studies on the isolated microalgae and cyanobacteria strains should address their plasticity towards salinity by thoroughly identifying the tolerance range, as well as the optimum salinity.

Moreover, the mechanisms of salt tolerance are closely linked to drought tolerance, a trait that is generally necessary for microalgae outside the aquatic environment, such as in a biocrust. Though there are some differences in the effect, as well as the protective mechanisms of these two stressors, common terrestrial microalgae that are already adapted to water deprivation have a good potential to withstand saline conditions as well. This is in congruence with our findings of several common terrestrial algae and cyanobacteria, such as *Pseudostichococcus* [119], *Bracteacoccus*, *Chloroidium* [68], and *Nodosilinea* [122], in biocrusts of potash tailing pile environments.

## 5. Conclusions

Many taxa of already known salt-tolerant green algae and cyanobacteria were found in biocrusts of extremely saline potash tailing pile habitats using an integrative approach. Several strains were the first record of that taxon in a biocrust. However, there was a high proportion of original isolates with unclear taxonomic positions, indicating new species or genera. Thus, biocrusts in potash tailing pile areas revealed unique communities of microalgae and cyanobacteria. Studies in other saline terrestrial habitats were often based on morphology, and new species could have been overlooked in these cases. Comparing our results to the previous morphological observations on the original isolates, our findings should be considered as an extension and improvement of the morphologically based species list. However, more detailed research is needed for deeper analyses of the newly found lineages. Consequently, our findings illustrated the benefits of an integrative approach but also underlined the limits of the molecular methods used in this study for describing new species.

The original stains examined in this study reflected a highly diverse and unique community of biocrust algae and cyanobacteria from potash tailing pile environments, which may be specialized for these extreme habitats. Their phylogenetic identification is an important but very first step in the ongoing research and will be crucial for the interpretation of subsequent eco-physiological experiments. Assessing the salt tolerance of the isolates is a necessary next step in understanding the ecology of microalgae and cyanobacteria in these specialized biocrusts. In the future, they may be of interest for the young field of applied biocrust research. Since biocrusts are multifunctional communities that are involved in several ecological processes, such as soil formation and surface stabilization, they could potentially be used to cover potash tailing piles [146]. The microalgae and cyanobacteria identified in this study represent candidates for artificial biocrust formation due to their origin in unique habitats close to the tailing piles and the high proportion of possibly new taxa.

## Figures and Tables

**Table 1:** Integrative species identifications compared to morphological identifications in a previous study [63]. Possibly undescribed taxa are marked (•). Additional strains that were not published and morphologically identified previously are placed in parentheses.

Original Strain	Integrated Approach	Morphological Approach [63]	Comments	Reference
Chlamydomonadales				
NN-4-1-N N-4-1-E	*Borodinellopsis texensis*	*Borodinellopsis texensis*	SSU rRNA sequences of the original strains were close to the authentic strain (SAG 17.95).	[104,105]
NN-2-N NN-2-D	*Borodinellopsis* sp. •	*Borodinellopsis* sp.	SSU rRNA sequences of the original strains formed a separate sister branch to the authentic strain of *B. texensis* (SAG 17.95).	[104,105]
WT-3-1-F	*Chloromonas* sp. •	*Radiosphaera negevensis*	SSU rRNA sequence of the original strain formed a separate lineage within *Chloromonas* together with the strain Ru-6-8.	[65,106,107,108]
NN-1-1-Q	cf. *Axilosphaera* •	*Tetracystis* sp.	SSU rRNA sequence of the original strain formed a separate clade close to *Axilosphaera* and *Eubrownia*.	[105]
SY-1-2-T TT-3-1-Q (TSN3f)	cf. *Chlorogonium* •	*Chlorococcum* sp. 1	SSU rRNA sequences of the original strains formed a separate lineage to *Chlorogonium.*	
SY-4-1-C TT-3-1-M	cf. *Spongiococcum* •	*Tetracystis* sp.	SSU rRNA sequences of the original strains formed a separate lineage within the Moewusinia.	[105]
(TTF-2-1-Da)	*Alvikia* sp. •		SSU rRNA sequences of the original strains formed a separate lineage within the *Alvikia*-clade.	[105]
Sphaeropleales				
TT-3-1-J TT-3-1-U NN-4-1-D2 NN-4-1-CC NN-4-1-H (TTF-1-1-M) (TTF-2-1-A) (TTF-2-1-J)	*Bracteacoccus minor*	*Bracteacoccus minor* *Bracteacoccus cohaerens* *Pseudomuriella aurantiaca*	SSU rRNA and ITS2 sequences of the original strains were close to the authentic strain (UTEX 66).	[109]
TT-3-1-G	*Tetradesmus dissociatus*	*Spongiochloris excentrica*	SSU rRNA and ITS sequences of the original strain were close to the authentic strain of *Tetradesmus dissociatus*.	[110,111]
Ulvophyceae				
TT-4-1-I	*Desmochloris* cf. *halophila* •	*Spongiochloris excentrica*	SSU rRNA and ITS sequences of the original strain were in an intermediate position between the authentic strain of *D. halophila* (CCAP 6006/1) and *D. mollenhaueri* (CCAP 6006/2), forming a separate branch.	[69,112,113]
TT-4-1-D TT-4-1-M TT-4-1-O NN-4-1-Q NN-4-1-S NN-4-1-T	*Halochlorococcum* sp. •	*Spongiochloris excentrica* *Spongiochloris* sp. *Chlorella* sp. 1 *Chlorococcum macropyrenoidosum*	SSU rRNA sequences of the original strains clustered in the clade formed by *Halochlorococcum* species, but the absence of a taxonomic revision of the genus did not allow identification of the strains to species level.	[114,115]
TT-3-1-I TT-4-1-J TT-4-1-CC TT-4-1-F (G2C)	*Planophila* sp. •	*Chlorella* sp. 2 *Chlorella* sp. 3 *Chlorella vulgaris* *Planophila* sp. 1	SSU rRNA and ITS sequences of the original strains formed a separate lineage within *Planophila*, distant from known species.	[69]
NN-4-1-B NN-4-1-M	cf. *Chlorothrix* •	*Ulothrix aequalis*	SSU rRNA and ITS sequences of the original strains formed a new lineage in the *Acrosiphonia*-clade of the Ulotrichales.	[113,116,117]
Trebouxiophyceae				
WT-3-1-L2	*Chlorella* cf. *pituita*	*Chlorella* sp. 2	SSU rRNA and ITS sequences of the original strain formed a separate branch from the authentic strain of *C. pituita* (ACOI 311).	[97]
OD-1-1-C	*Chloroidium saccharophilum*	*Chloroidium ellipsoideum*	SSU rRNA and ITS sequences of the original strain were close to the authentic strain (SAG 211-9a).	[68,118]
OD-1-2-N OD-1-1-K SY-1-2-B NN-2-O	*Chloroidium* sp. •	*Chloroidium ellipsoideum*	SSU rRNA and ITS sequences of the original strains fell on separate branches from *C. lichinum* and *C. ellipsoideum*.	[68,118]
SY-1-2-K	*Diplosphaera chodatii*	*Diplosphaera chodatii*	SSU rRNA and ITS sequences of the original strain were close to the authentic strain (SAG 48.86).	[119]
TT-2-1-CA1 TT-3-1-F NN-4-1-D (TTF-2-1-D)	*Nannochloris* sp.	*Nannochloris* sp.	SSU rRNA sequences of the original strains fell in two separate lineages within *Nannochloris*-like algae. No revision has addressed the polyphyletic positions of *Nannchloris*.	[101,120]
TT-4-1-S (TSN1) (TSN3)	*Pseudochlorella signiensis*	*Parietochloris* cf. *cohaerens*	SSU rRNA and ITS sequences of the original strains were close to the authentic strain of *P. signiensis* (SAG 7.90).	[121]
TT-4-1-K OD-1-1-X WT-3-1-P1 WT-3-1-A SY-1-2-P WT-3-1-H NN-1-1-X (TTF-3-1-BB)	*Pseudosticho- coccus * *monallantoides*	*Stichococcus bacillaris* *Stichococcus exiguus* *Stichococcus allas*	SSU rRNA and ITS sequences of the original strains were close to the authentic strain of *P. monallantoides* (SAG 380-1).	[119]
NN-2-1-X1B OD-1-1 NN-1-1-E NN-1-1-M NN-1-1-L	*Watanabea* sp.	*Parietochloris cohaerens* *Parietochloris* cf. *ovoideus*	SSU rRNA and ITS sequences of the original strains formed a separate lineage within *Watanabea*.	[70]
Cyanobacteria				
SY-1-2-EE SY-1-2-Y	*Cyanocohniella* sp.	*Nostoc* sp.	SSU rRNA and ITS sequences of the original strains formed separate lineages close to *Cyanocohniella*.	[75,76]
OD-1-1-T SY-4-1-H	*Nodosilinea bijugata*	*Leptolyngbya* sp.	SSU rRNA and ITS sequences of the original strains were close to the authentic strain of *N. bijugata* (Kovacik 1986/5a).	[122]
NN-2-1-EE NN-3-1-CD TT-1-1-CA	*Nodosilinea* cf. *signiensis*	*Leptolyngbya* sp.	SSU rRNA and ITS sequences of the original strains were close to the authentic strain of *N. signiensis* (USMFM), but the clade thus formed was not statistically supported.	[123]
OD-2-1-CH	*Nodosilinea* sp. •	*Leptolyngbya* sp.	SSU rRNA and ITS sequences of the original strain formed a separate lineage with the unidentified strain KIOST-1.	[122]
NN-4-1-FF NN-3-1-CA NN-3-1-G NN-4-1-O	“*Phormidesmis*” sp. •	*Pseudophormidium edaphicum* Cyanobacteria 2 Cyanobacteria 5	Unclear genus: no revision for the clade “*Phormidesmis*” present	[72]
NN-3-1-B NN-3-1-D	“*Pseudophormidium*” *battersii* •	Cyanobacteria 5 Cyanobacteria 2	Unclear genus: SSU rRNA sequences of the original strains formed a separate clade together with the strain KZ-16-2 previously identified as *“Pseudophormidium*” *battersii*. The group requires revision.	[72]

## Supplementary Materials

The following are available online at https://www.mdpi.com/2076-2607/8/11/1667/s1: Table S1: Gene Bank Accession Number and origin/habitat description of the reference strains used for phylogenetic tree construction.
