# Supplementary material for: Uncovering Unique Green Algae and Cyanobacteria Isolated from Biocrusts in Highly Saline Potash Tailing Pile Habitats, Using an Integrative Approach
Source: Microorganisms. 2020 Oct 27;8(11):1667. doi: 10.3390/microorganisms8111667 (PMC7692164; doi:10.3390/microorganisms8111667)
Supplement: Supplementary file 1 [file microorganisms-08-01667-s001.pdf]

Table S1 Gene Bank Accession Number and origin/habitat description of the reference strains used for phylogenetic tree construction

Gene Bank Nr.	Taxon	Strain	Origin/Habitat
**MK211230**	*"Pseudophormidium" battersii*	KZ-16-2	Ukraine: the Crimea, Leninsky Distinct, Kazantip national reserve, chasmoendoliths, limestone outcrops, coast of Azov Sea, coast of Azov
**AJ626846**	*"Urospora"* sp. uncultured		Antarctica: Terra Nova Bay, sea water
**U63099**	*Bracteacoccus giganteus*	UTEX 1251	USA
**KF673367**	*Bracteacoccus minor*	SAG 221-1	soil
**JQ281841**	*Bracteacoccus ruber*	CCAP 221/7	Antarctica: Mt Erebus, Ross Island, soil
**MK231272**	*Bracteacoccus xerophilus*	KZ-2-2-6	Ukraine: the Crimea, Leninsky Distinct, Kazantip national reserve, biocrust, clay scree, coast of Azov
**FR865616**	*Chlamydomonas applanata*	CCAP 11/9	Czech Republic: botanic garden, Prague University, decaying leaf litter
**FR865582**	*Chlamydomonas dorsoventralis*	CCAP 11/4	Czech Republic: Hirschberg, pond, Dorfteich
**JQ315503**	*Chlamydomonas hedleyi*	KMMCC 188	seawater
**FR865590**	*Chlamydomonas leicostraca*	CCAP 11/49	UK: Cambridge, England, Madingley Road, farmyard puddle
**AB511834**	*Chlamydomonas reinhardtii*	UTEX 90	USA: Amherst, Massachusetts, potato field
**JQ315516**	*Chlamydomonas* sp.	KMMCC 189	seawater
**GQ176853**	*Chlorella pituita*	ACOI 311	Portugal: Mira, trout nursery
**MH415449**	*Chloroidium lichenum*	Vancurova OP1118.1	Czech Republic: lichen thallus, slag, on *Stereocaulon nanodes*
**FR865528**	*Chloromonas rosae*	CCAP 11/112	Norway: østfold, Lake Hollitjernet (freshwater)
**MH703736**	*Chloromonas* sp.	Ru-6-8	Germany: Prora, Ruegen, biocrust, sand dunes, Baltic Sea coast
**AY476827**	*Chlorothrix* sp.	ChloPac47SI	Canada: Ucluelet, Vancouver I., British Columbia
**KM020111**	*Coccobotrys mucosus*	SAG 24.92	Antarctica: Princess Elizabeth Land, Vestfold Hills, nutrient enriched soil, elephant seal wallow
**KM020110**	*Coccobotrys verrucariae*	SAG 16.97	Switzerland: phycobiont of lichen *Verrucaria* spec.
**FM882216**	*Desmochloris halophila*	CCAP 6006/1	Africa
**FM882217**	*Desmochloris mollenhaueri*	CCAP 6006/2	South Africa: Flaminkvlakte, western Succulent Karoo, semi-desert, biocrust
**LT560371**	*Edaphochlorella mirabilis*	SAG 211-30	Germany: Göttingen, Old Botanical Garden University of Göttingen, basin
**FJ648518**	*Elliptochloris subsphaerica*	SAG 2202	Japan: Yokogawa, Hiroshima-City, on surface of bark

**MH703757**	*Emerochloris sphaerica*	Us-s-7-5	Germany: Karlshagen, Usedom, biocrust, Baltic Sea coast
**FR865661**	*Graesiella emersonii*	CCAP 211/5	UK: England, freshwater
**AM412750**	*Koliella sempervirens*	CCALA 363	Slovakia: Bratislava
**HE984579**	*Leptochlorella corticola*	I2e	Slovenia: Portoroz, bark of *Cupressus sempervirens*
**KF693810**	*Leptochlorella* sp.	UTEX EE84	Israel: Negev Desert
**FR798945**	*Leptolyngbya foveolarum*	VP1-08	Italy: Firenze, Villa La Pietra, concrete-made fountain with stagnant water, gray dry crust
**KP081398**	*Myrmecia cf. irregularis*	LH08AW3064	Germany: terrestrial
**JQ922411**	*Nannochloris* sp.	AICB 424	
**MH305359**	*Nannochloris* sp.	Z5	Russia: Siberia, saline lake Shira
**HM018677**	*Nodosilinea epilithica*	Kovacik 1998/7*	Italy: Vieste, Foggia, Peninsula Gargano, green biofilm on the wall of house
**KJ004406**	*Phormidesmis* sp.	ACL_P2D9	USA: Oroville, Washington, microbial mat in hypersaline hot lake
**MF034636**	*Planophila bipyrenoidosa*	ULVO-1	alpine chalk soils?
**AY493583**	*Plectolyngbya hodgsonii*	ANT.LPR2.2	Antarctica: Long peninsula, saline Pendant lake
**X91268**	*Pseudomuriella aurantica*	SAG 249-1	Switzerland: Basel, Ritterstrasse 31, wet shaded tuff in garden
**AB360750**	*Rusalka fusiformis*	NIES-123	Japan: Niseko, Hokkaido, Lake Shinsen-numa (freshwater)
**KM020016**	*Tetracystis* sp.	14601-7.1	
**MH703773**	*Tetradesmus arenicola*	WD-7-1	Germany: Warnemuende, sand dunes, Baltic Sea coast, biocrust
**MG022741**	*Tetradesmus obliquus*	CCAP 276/3C	Germany: Marburg, freshwater
**DQ821515**	*Ulothrix* sp.	X1	USA: west shore of Amaknak Island, Alaska, mid intertidal boulders near the Dutch Harbor airport
**DQ821516**	*Ulothrix* sp.	X3	USA: west shore of Amaknak Island, Alaska, mid intertidal boulders near the Dutch Harbor airport
**FN811228**	uncultured cyanobacterium	UMAB-cl-44	Antarctica: Alexander Island, soil
**AY476821**	*Urospora neglecta*	strain Seward 8	Canada: Seward, Alaska
**AJ626846**	*Urospora* sp.	uncultured	Antarctica: Terra Nova Bay, the Italian Antarctic Station, thick green mats on granitic rocks
**MH827565**	*Watanabea acidotolerans*	CAUP H 8901	Czech Republic: stone surface, found at the bottom near shore of acidic (pH 2.6) Hromnice Lake
**MH499911**	*Watanabea borysthenica*	SAG 2550	Ukraine: Trakhtemyriv Nature Reserve, on surface of sandstone
**KC463197**	*"Leptolyngbya" subtilissima*	EcFYyyy700	South Africa: Succulent Karoo, semi-desert, biocrust
**JX513885**	*"Scotiellopsis" reticulata*	CCALA 474 2	Romania: Constanta, sand, psammon
**KY807917**	*„Chlorogloea“ microcystoides*	SABCo22904	Ireland: Kilkee
**AY455943**	*Acrosiphonia coalita*		USA: California, Humboldt County, Baker Beach
**HE610119**	*Acrosiphonia* sp.	SAG 127.80	Germany: Helgoland, marine

**FN562430**	*Acrosiphonia* sp.	SAG 127.80	Germany: Helgoland, marine
**AB049418**	*Acrospiphonia duriuscula* var. *tenuis*		
**MH703777**	*Actinochloris sphaerica*	SAG 23.93	Germany: Brandenburg, Güterfelde/Potsdam, loamy sandy soil from winter barley field
**AB936278**	*Actinochloris sphaerica*	UTEX 121	
**AY510463**	*Acutodesmus deserticola*	BCP-YPGChar	USA: Yuma Proving Ground, Arizona
**MK503792**	*Aliinostoc catenatum*	SA24	Iran: Mazandaran province of Iran, garden soil
**MK503793**	*Aliinostoc constrictum*	SA30	Iran: Mazandaran province of Iran, paddy field
**NR_158066**	*Aliinostoc morphoplasticum*	MCC3177	India: Sihora, Jabalpur, eutrophic pond
**AB058336**	*Alvikia littorale*	MBIC10280	
**JX169829**	*Apatococcus lobatus*	SAG 2151	Germany: Rostock, Südstadt, Max-Planck-Str., on plastic switchbox
**JX169826**	*Apatococcus lobatus*	SAG 2359	Germany: Göttingen, Obere Karspüle 18, on green plastic biowaste bin
**KM020133**	*Axilosphaera vegetata*	SAG 30.95	USA: TN, Wilson Country, surface soil from cedar glades of Lebanon State Forest
**AJ000206**	*Blidingia minima*		UK: Scotland
**AY303596**	*Bolbocoleon piliferum*	WAz1	USA: Cattle Point, San Juan Island, Washington, endophytic in *Cymathere triplicata*
**MF101222**	*Borodinellopsis texensis*	JM002	Thailand: sea salt farm
**KM020129**	*Borodinellopsis texensis*	SAG 17.95	USA: Texas, soil from Mustang Islan
**JQ281839**	*Bracteacoccus aerius*	UTEX 1250	USA: Austin, Texas, air-borne dust
**JQ281840**	*Bracteacoccus aggregatus*	G2-3	Germany: Göttingen, meadow
**MH703740**	*Bracteacoccus aggregatus*	Ru-s-4-5	Germany: Prora, Ruegen, biocrust, sand dunes, Baltic sea coast
**MH703758**	*Bracteacoccus aggregatus*	Us-s-7-3	Germany: Zempin, Usedom, biocrust, baltic sea coast
**JF717415**	*Bracteacoccus bohemiensis*	KF-2011b	Czech Republic
**JQ281848**	*Bracteacoccus bullatus*	SAG 2032	Germany: near Harz mountains, soil from mining dump, contaminated with heavy metals
**JQ259931**	*Bracteacoccus bullatus*	SAG 2317	
**JQ281857**	*Bracteacoccus deserticola*	BCP-EM3-VF7	USA: Sierra San Pedro Martir of Baja California, Mexico, decomposed granite sandy soil
**HQ246427**	*Bracteacoccus giganteus*	UTEX 1252	USA: Enchanted Rock, Texas
**JQ281862**	*Bracteacoccus glacilis*	Broady 686	Antarctica
**JF717398**	*Bracteacoccus minor*	UTEX 66	
**JQ259951**	*Bracteacoccus occidentalis*	BCP-WJT8-VFNP 19	USA: Joshua Tree National Park, California
**JQ281867**	*Bracteacoccus polaris*	KF28	Sweden: Abisko

**HQ246424**	*Bracteacoccus pseudominor*	UTEX 1247	
**AF516676**	*Bracteacoccus* sp.	BC2-1	USA: desert soil
**JQ281873**	*Bracteacoccus xerophilus*	BCP-ZNP1-VF32	USA: Utah, Zion National Park
**EF490447**	*Brasilonema terrestre*	CNA116	
**NR_114995**	*Calothrix desertica*	PCC 7102	Chile: Antofagasta, sand
**HF678479**	*Calothrix parietina*	CCAP 1410/10	UK: England, Durham, freshwater stream
**KU951670**	cf. *Phormidesmis* sp.	LEGE 11434	Portugal: less than 1 km off the shore, near Leixoes Harbour, subtidal
**MF034630**	*Chamaetrichon brasiliensis*	SAG 1.87	USA: TE, Nashville, pool at Couchville Pike
**MF034631**	*Chamaetrichon brasiliensis*	SAG 2396	Germany: western margin of the Franconian Alb, tufa-forming karstwater stream "Deinschwanger Bach", biofilm on rock surface
**JN385292**	*Champylonemopsis* sp.	HA4241-MV5	USA: Oahu, Hawaii, Laie Falls
**LC322160**	*Chlamydomonas bilatus*	SAG 7.72	Slovakia: High Tatra Mountains, pool (freshwater)
**FR865606**	*Chlamydomonas hydra*	CCAP 11/6B	freshwater
**JN903986**	*Chlamydomonas macrostellata*	SAG 72.81	France: Jardin Alpin du Museum, Paris, freshwater ditch
**AB058373**	*Chlamydomonas parkeae*	MBIC10599	
**AJ781313**	*Chlamydomonas raudensis*	CCAP 11/131	Antarctica: Lake Bonney
**GU117583**	*Chlamydomonas reticulata*	CCCryo 154-01	Norway: Svalbard, High Arctic, snow, from the surface of the Svenbreen glacier
**KP313859**	*Chlamydomonas* sp.	UWO 241	Antarctica: Lake Bonney
**HQ111432**	*Chlorella chlorelloides*	CB 2008/110	Germany: Mecklenburg Vorpommern, plankton in lake Pragsdorfer See (freshwater)
**FM205862**	*Chlorella coloniales*	UTEX 938	
**FM205858**	*Chlorella elongata*	CCAP 211/18	
**FM205861**	*Chlorella lewinii*	CCAP 211/90	Chile: Easter Island, soil from the edge of a permanent freshwater pond in a crater
**FM946019**	*Chlorella lichenum*	SAG 2115	Germany: Göttingen, Nikolausberger Weg 18, on silicone insolation
**FM205833**	*Chlorella lobophora*	SAG 37.88	former USSR:, Briamskaia district, Krasmyj Rog, soil from mixed forest
**FM205856**	*Chlorella pituita*	ACOI 856	Portugal: Serra da Estrela, Manteigas, flowing water
**HQ111431**	*Chlorella pulchelloides*	CCAP 211/118	Germany: Feldberger Haussee (freshwater)
**HQ111433**	*Chlorella rotunda*	CCAP 260/11	Angola: Okawango, freshwater
**KM020042**	*Chlorella salina*	SAG 8.86	UK: North Wales, oyster breeding tank at Conway
**HQ111435**	*Chlorella singularis*	CCAP 211/119	Kenya: Nakuru Sewage Pond, freshwater
**FM205834**	*Chlorella sorokiniana*	SAG 211.8k	

**Y14950**	*Chlorella* sp.	Yanaqocha RA1	Peru: Lake Yanaqocha?
**HQ111434**	*Chlorella volutis*	CCAP 211/120	Kenya: Nakuru National Park, Rhinopool, freshwater
**FN298917**	*Chlorella vulgaris*	CCAP 211/109	USA: endosymbiont of Paramecium bursaria, freshwater
**FR865659**	*Chlorella vulgaris*	CCAP 211/11Q	former Czechoslovakia: freshwater
**AY591508**	*Chlorella vulgaris*	SAG 211-11b	Netherlands: eutrophic pond near Delft
**MH703759**	*Chlorella vulgaris*	Us-s-7-4	Germany: Zempin, Usedom, biocrust, Baltic Sea coast
**AB058302**	*Chlorococcum dorsiventrale*	MBIC10044	
**FR865693**	*Chlorocystis* sp.	CCAP 233/1	France: Soulac, from *Ulva* culture
**MK541803**	*Chlorocystis* sp.	CCAP 6005/13	Madagascar: Chenal d'Ampanarata , Belo sur Mer, epiphyte on *Bostrychia pinnata* (marine)
**AB278611**	*Chlorogonium capillatum*	NIES-692	Japan: Miyatoko Mire Fukushima Japan, freshwater bog
**AB278621**	*Chlorogonium elongatum*	NIES-751	UK: England, Caldbeck., freshwater pond
**AB278607**	*Chlorogonium euchlorum*	UTEX 2010	Germany
**KR607488**	*Chlorogonium* sp.	SAG 17	
**MH551519**	*Chloroidium antarcticum*	ISBAL-103	Antarctica: South Shetlands, King George Island, Ecology Glacier
**MH551521**	*Chloroidium arboriculum*	MG-3	Vienna: Vienna Forest, bark of tree, free-living biofilm on *Fagus sylvatica*
**FM946012**	*Chloroidium ellipsoideum*	SAG 3.95	
**FM946015**	*Chloroidium ellipsoideum*	SAG 2143	Germany: Rostock, Südstadt, Ernst-Häckel-Str.12-15, east, on rough plaster
**FM946011**	*Chloroidium engadinense*	SAG 812-1	Switzerland: Unterengadin, soil
**HF586465**	*Chloroidium laureanum*	CAUP H8501	Slovenia: Ankaran, corticolous microbial biofilms, bark of *Laurus nobilis*
**FM946020**	*Chloroidium lichenum*	SAG 2144	Germany: Rostock, Südstadt, Brahestr. 7-11, west, on rough plaster
**MH551523**	*Chloroidium lobatum*	CAUP 8502	Slovenia: Pacug, bark of *Fraxinus ornus*
**FM946000**	*Chloroidium saccharophilum*	SAG 211-9a	Germany: sap from wounded *Populus alba*
**MH703747**	*Chloroidium* sp.	Ru-6-6	Germany: Prora, Ruegen, biocrust, sand dunes, Baltic Sea coast
**FR865615**	*Chloroidium* sp.	CCAP 11/8	former Czechoslovakia: freshwater
**MH415214**	*Chloroidium* sp.	LV-2018	Czech Republic: Dobany, lichen thallus (*Stereocaulon vesuvianum*), on industrial foam
**MH551495**	*Chloroidium viscosum*	SAG 56.87	Austria: Burgenland near Tatzmannsdorf, trunk of *Quercu*s, phycobiont of lichen *Woessia fusarioides*
**AJ410445**	*Chloromonas actinochloris*	SAG 1.72	USA: Texas, Caldwell Co., soil
**MG189707**	*Chloromonas arctica*	CCALA:10278	Norway: Svalbard, High Arctic, snow, from the surface of the Svenbreen glacier
**AJ410452**	*Chloromonas aufgustae*	SAG 5.73	former Czechoslovakia: mucous covers in swamp, freshwater
**AY653740**	*Chlorothrix sp.*	C167	Canada: Ucluelet, Vancouver, British Columbia

**MK211229**	*Chroakolemma edaphica*	KZ-23-2	Ukraine: the Crimea, Leninsky Distinct, Kazantip national reserve, biocrust, clay scree, coast of Azov
**GU827477**	*Chromochloris zofingiensis*	CAUP H6503	Sweden: Lake Erken
**KF673375**	*Chropmochloris zofingiensis*	SAG 221-2	Switzerland: Unterengadin, soil
**HG972998**	*Coccomyxa dispar*	SAG 49.84	Germany: peat soil in Steinhauser Ried, phycobiont of lichen *Multiclavula vernalis*
**HG973006**	*Coccomyxa viridis*	SAG 2325	France: Tours, from protoplast cell cultures of a *Gingko biloba* tree from botanical garden
**JX513883**	*Coelastrella* sp.	SAG 2123	Germany: Schermbeck, Haus Dr. Ammenwerth, Burgstrasse, edge of the woods, rooftile
**AF388376**	*Coelastrum sphaericum*	SAG 32.81	Hungary: freshwater
**JX169834**	*Coenochloris signiensis*	CCAP 176/3	Antarctica: Signy Island, South Orkney Islands, Antarctica, soil and moss
**NR125521**	*Coleofasciculus chthonoplastes*	strain SAG 2209	Germany: Zingst, Baltic Sea, wind flat, microbial mat
**MF034603**	*Ctenocladus circinnatus*	CCMP 2158	sandstone
**MF034606**	*Ctenocladus circinnatus*	ULVO-18	sandstone
**KJ737428**	*Cyanocohniella calida*	CCALA 1049	Czech Republic: Karlovy Vary, hot thermal spring
**MN243143**	*Cyanocohniella crotaloides*	PJ S45	Netherlands: Schirmonnikoog Island, cyanobacterial mats
**MT425938**	*Cyanocohniella* sp.	ACSSI 322	Russia: soil
**FR774768**	*Cyanopsira rippkae*	NMBCI	Chad: Eastern fringe of Lake Chad, closed hyperalkaline pond
**AY038036**	*Cyanospira rippkae*	PCC 9501	Kenya: hypersaline soda lake Magadi
**KF144190**	*Desmochloris* cf. *halophila*	SAG 2397	Germany: Franconian Alb, Deinschwanger Creek, biofilm on rock surface in karstwater stream
**AB049416**	*Desmochloris halophila*	UTEX 2073	brakish water
**MH703754**	*Desmochloris halophila*	Ru-s-3-2	Germany: Rügen, Glowe, biocrust, sand dunes, Baltic Sea coast
**FM882218**	*Desmochloris mollenhaueri*	CCAP 6006/3	South Africa: Flaminkvlakte, western Succulent Karoo biome, semi-desert, biocrust
**KY086483**	*Desmochloris* sp.	ACSSI 180	Russia: Oblast Wolgograd
**KF791549**	*Desmochloris* sp.	JB13	China: Songnen Plain, saline-alkali soil, grassland
**AJ431571**	*Desmococcus endolithicus*	SAG 15.92	Antarctica: Edward VII Peninsula, Marie Bird Land, chasmoendolithic at Scott Nunataks
**KM020049**	*Desmococcus olivaceus*	SAG 1.92	Austria: Vienna, bark
**KP726266**	*Desmodesmus denticulatus*	KLL-G003 clone	Israel
**AM711523**	*Desmonostoc mucosum*	Lukesova 1/87	Czech Republic :Plzensky kraj, Dlouha Ves, soil sample from wet meadow
**HG004585**	*Desmonostoc* sp.	PCC 9230	Canada: Park 1, Halifax, Nova Scotia
**KM020081**	*Dictyochloris fragrans*	SAG 220-1b	Switzerland: soil from National Park Unterengadin at II Fuorn
**GU017656**	*Dictyochloropsis splendida*	HEW11-R2	Germany: bark of beech tree

**GU017649**	*Dictyochloropsis splendida*	SAG 2071	Japan: Nishi-kawaguchi-cho, Hiroshima-Pref., on surface of bark
**FM205840**	*Didymogenes palatina*	SAG 30.92	Germany: Jülich, fire fighters pond at KFA
**MF124975**	*Diplosphaera chodatii*	D52	Canada: Manitoba, Payuk Lake
**MK201746**	*Diplosphaera chodatii*	KZ-26-5	Ukraine: the Crimea, Leninsky Distinct, Kazantip national reserve, chasmoendoliths, limestone outcrops, coast of Azov Sea, coast of Azov
**MT078177**	*Diplosphaera chodatii*	SAG 11.88	New Zeeland: Waweira Scenic Reserve, phycobiont of lichen *Pseudocyphellaria carpoloma* on *Rhopalostylus sapida*
**MT078178**	*Diplosphaera chodatii*	SAG 48.86	Antarctica: Princess Elizabeth Land, Vestfold Hills, from moss
**HQ129931**	*Diplosphaera chodatii*	UTEX 1177	USA: Texas, Blackland Prairie, soil
**KM020116**	*Diplosphaera chodatii*	SAG 49.86	Switzerland: Basel, Botanical Garden University Basel, water bloom small bog
**MH703748**	*Diplosphaera* sp.	Ru-6-16	Germany: Prora, Ruegen, biocrust, sand dunes, Baltic Sea coast
**DQ009763**	*Dunaliella parva*	SAG 19-1	Romania: Lacul Sarat, marine
**DQ530055**	*Elliptochloris bilobata*	SAG 245.80	Austria: Kärnten, Kreuzeckgruppe, 2200 m, phycobiont of *Catolechia wahlenbergii*
**AJ005413**	*Enteromorpha intestinalis*	SY0104	
**AY510465**	*Flechtneria rotunda*	BCP-SEv3-VF49	USA: Sevilleta LTER, Socorro Co., New Mexico, soil
**HQ847570**	*Fortiea* sp.	HA4221-MV2	USA: Oahu, Hawaii, Laie Falls
**AM412753**	*Gloeotila scopulina*	SAG 335-8	Sweden: freshwater
**AB360742**	*Gungnir kasakii*	CCAP 12/8	UK: Priest Pot, Cumbria, England, freshwater
**AB360747**	*Haematococcus lacustris*	NIES-144	Japan: Sapporo Hokkaido Japan, freshwater lake
**KM020176**	*Halochlorococcum dilatum*	SAG 12.90	Germany: Helgoland, rock-pool
**AY198122**	*Halochlorococcum moorei*	Wa14-B	USA: San Juan Island, endophyte in *Blidingia* cf. *minima*
**DQ821520**	*halochlorococcum porphyrae*		USA: California, Carmel, endophyte in *Porphyra schizophylla*, marine
**MF034634**	*Halofilum salinum*	SAG 1.95	France: schorre de estuary of river Orne at Sallenelles, from piece of wood
**MF034615**	*Halophilum ramosum*	SAG 2050	UK: Anglesey, Porth Trecastel, in stonecracks of coastal rocks, phycobiont of *Wahlenbergiella striatula*
**KJ843313**	*Halotia longispora*	CENA420	Antarctica: Baranowski glacier biofilm growing on rocks, saline
**KC695854**	*Halotia wernerae*	CENA 160	Brazil: mangrove, Cardoso Island, saline soil
**AM905327**	*Hassallia byssoidea*	CCALA 823	Czech Republic: South Moravia, cyanobacterial mat on granitic rock
**KM020117**	*Heterochlamydomonas inaequalis*	SAG 4.75	USA: Tennessee, forest soil from Wilson Co.
**LC057289**	*Ixipapillifera sacculiformis*	SAG 63.72	Germany: Lüneburger Heide, freshwater pond

**AY493607**	*Leptolyngbya antarctica*	ANT.L18.1	Antarctica: Lake Spate/Oskar, Larsemann Hills, microbial mat
**HF678483**	*Leptolyngbya boryana*	UTEX B 488	
**KX765291**	*Leptolyngbya ectocarpi*	SABC012402	Ireland: Ballybunion
**FM210757**	*Leptolyngbya laminosa*	ETS-08	Italy: Padova, Montegrotto Terme, Euganean hot thermal springs, microbial mats
**FR798934**	*Leptolyngbya margaretheana*	1T12	Italy: Firenze, Piazza SS. Annunziata, Tacca's fountain, green biofilm under the water flow on the marble pedestal
**AY493584**	*Leptolyngbya* sp.	ANT.L52.1	Antarctica: Brocknes, Bruehwiler lake (freshwater), microbial mat
**GQ859652**	*Leptolyngbya tenuis*	PMC304.07	France
**Z68696**	*Leptosira erumpens*	UTEX 979	USA: McMahan, Texas, Carrizo Sands
**Z68695**	*Leptosira obovata*	SAG 445-1	Switzerland: Basel, boggy water at Rosenau
**U70594**	*Lobochlamus culleus*	SAG 18.72	USA: Florida, field near Maxville
**AJ410456**	*Lobochlamus segnis*	SAG 52.72	Czech Republic: Altvater-Gebirge, soil from beech forest near Winkelsdorf
**AJ410461**	*Lobochlamys culleus*	SAG 17.73	Czech Republic: pond in Nordmähren (freshwater)
**KM020046**	*Lobosphaera incisa*	SAG 2466	
**AB080301**	*Marvania coccoides*	CCAP 251/1b	UK: Cambridge, England, freshwater
**KM020037**	*Marvania geminata*	SAG 12.88	former Czechoslovakia: freshwater
**FR854374**	*microglena basinucleata*	SAG 67.72	Germany: Göttingen, University, pond in Botanical Garden
**AY577534**	*Mojavia pulchra*	JT2-VF2	USA: Joshua Tree National Park, California, desert soil
**KJ939052**	*Myxacoris californica*	WJT36-NPBG1	USA: Joshua Tree National Park, Wonderland of Rocks, San Bernardino Co., California, granitic desert soil
**KJ939079**	*Myxacoris chilensis*	ATA2-1-KO14	Chile: biocrust in Atacama Desert, desert soil
**AB080303**	*Nannochloris atomus*	CCAP 251/7	marine
**AB080300**	*Nannochloris bacillaris*		
**AB080302**	*Nannochloris maculata*	CCAP 251/3	marine
**AY195983**	*Nannochloris* sp.	JL 4-6	USA: Arrowood National Wildlife refuge, North Dakota, Jim Lake (freshwater)
**MK231273**	*Nannochloris* sp.	KZ-2-2-4	Ukraine: the Crimea, Leninsky Distinct, Kazantip national reserve, biocrust, clay scree, coast of Azov
**MK231273**	*Nannochloris* sp.	strain KZ-2-2-4	Ukraine: the Crimea, Leninsky Distinct, Kazantip national reserve, biocrust, clay scree, coast of Azov
**JQ920362**	*Neocystis brevis strain*	CCALA 393	Switzerland: Unterengadin, soil

**JQ920367**	*Neocystis mucosa*	SAG 40.88	Antarctica: freshwater
**AJ416104**	*Neodangemannia microcystis*	SAG 2022	Germany: Helgoland, on old shell in tide pool
**AB936280**	*Neospongiococcum proliferum*	UTEX 1846	USA: Memphis, Tennessee, USA. cotton field, Shelby Forest
**EU528669**	*Nodosilinea bijugata*	str. KOVACIK1986/5a	Poland: littoral region Lake Piaseczno
**MH688852**	*Nodosilinea* cf. *epilithica*	Ru-6-11	Germany: Prora, Ruegen, biocrust, sand dunes, Baltic Sea coast
**MH688851**	*Nodosilinea* cf. *epilithica*	Us-2-1	Germany: Karlshagen, Usedom, biocrust, Baltic Sea coast
**KF307598**	*Nodosilinea nodulosa*	UTEX 2910	China: Hong Kong, South China Sea, ocean plankton
**MF352005**	*Nodosilinea radiophila*	TM S2B	Iran: thermal springs Ramsar, Mazadaran Province, soil samples, high radioactivity
**MF348321**	*Nodosilinea ramsarensis*	KH-S S2.6	Iran: Ramsar ,Mazandaran Province, soil around the thermal spring (32 °C)
**AJ639893**	*Nodosilinea* sp.	0BB24S04	Italy: Emilia-Romagna, Imola, Bubano Basin
**HM217061**	*Nodosilinea* sp.	LEGE 073141	Portugal: Douro estuary, Douro-Porto edge
**KM019929**	*Nodularia harveyana*	SAG 44.85	UK: Lincolnshire, salt marsh near Gibraltar Pt., brackish water
**AF268022**	*Nodularia spumigena*	UTEX B2092	Canada: Osoyoos, British Columbia, Canada, alkaline soil
**HM018687**	*Oculatella coburnii*	WJT36-NPbg13	USA: Joshua Tree National Park, California
**EU528672**	*Oculatella neakameniensis*	str. Kovacik 1990/54	Greece: Nea Kammeni Island, Volcano
**AB183610**	*Oltmannsiellopsis geminata*	MBIC10525	Endophytic in *Cymathere triplicata*
**FN562431**	*Oltmannsiellopsis viridis*	NIES 360	Japan: Onagawa Bay Miyagi
**AJ410466**	*Oogamochlamys gigantea*	SAG 9.84 *	USA: Texas, Llano Co., soil from Enchanted Rock
**EU878373**	*Parietochloris alveolaris*	UTEX 836	
**MF034625**	*Paulbroadya pertersii*	ULVO-34	UK: Oban, Scotland, phycobiont isolated from the lichen *Verrucaria mucosa*
**FR865730**	*Pectinodesmus pectinatus*	CCAP 276/51	Finnland: Långkär, Tvärminne, freshwater
**AY454429**	*Phaeophila dendroides*	MA2.4a1	USA: Quissett Estuary, Woods Hole, MA, in oyster shell
**KU219737**	*Phormidesmis arctica*	MUM 11-7 CCALA 1101 *	Norway: Svalbard, Petunia Bay
**AY493581**	*Phormidesmis priestleyi*	ANT.L66.1	Antarctica: Larsemann Hills, microbial mat in freshwater lake
**AY493586**	*Phormidesmis priestleyi*	ANT.LACV5.1	Antarctica: Long peninsula, Vestfold Hills, saline Ace lake, microbial mat
**AY493620**	*Phormidesmis pristleyi*	ANT.LPR2.5	Antarctica: Long peninsula, Vestfold Hills, saline Pendant lake, microbial mat
**KT731154**	*Phormidesmis* sp.	CENA335	Brazil: Bertioga/SP, leaf of *Avicennia schaueriana* (mangrove)

**KT731156**	*Phormidesmis* sp.	CENA339	Brazil: Bertioga/SP, leaf of *Avicennia schaueriana* (mangrove)
**AY493627**	*Phormidium murrayii*	ANT.LACV5.2	Antarctica: Long peninsular, Vestfold Hills, saline Ace lake, microbial mat
**GQ859651**	*Phormidium* sp.	PMC301.07	
**AY526738**	*Picochlorum* sp.	RCC115	marine
**AY422076**	*Picochlorum* sp.	UTEX 2378	marine
**MF034637**	*Planophila bipyrenoidosa*	ULVO-55	alpine chalk soils?
**MF034638**	*Planophila laetevirens*	SAG 2008	Italy: South Tyrol, Italy, Dolomites, soil
**MF034642**	*Planophila laetevirens*	SAG 465-1	Switzerland: Basel, garden pool
**MF034640**	*Planophila laetevirens*	ULVO-56	Ukraine: Cherkassy Oblast, Regional Landscape Park „Trakhtemyriv“, sandstone, chasmoendolithic
**MF034618**	*Pseudendoclonium commune*	SAG 2236	Sweden: Öland, Baltic sea, phycobiont isolated from the lichen *Verrucaria maura*
**MF034610**	*Pseudendoclonium incrustans*	CCAP 415/1	Austria: phycobiont in lichen *Verrucaria aquatilis*
**MF034619**	*Pseudendoclonium submarinum*	SAG 2237	UK: West Scotland, phycobiont isolated from the lichen *Verrucaria maura*(?)
**X63520**	*Pseudochlorella pringsheimii*	SAG 211-1a	freshwater
**LT560358**	*Pseudochlorella pyrenoidosa*	SAG 18.95	Italy: South Tyrol, soil from Dolomites
**KM116464**	*Pseudochlorella signiensis*	SAG 7.90	Antarctica: South Orkney Islands, Signy Island, soil
**LT560366**	*Pseudochlorella signiensis*	PS-2	Ukraine: Island Zmijiny, Black Sea, soil
**KM116465**	*Pseudochlorella signiensis*	SAG 2110	Göttingen: Nikolausberger Weg 18, from roof tile
**LT560361**	*Pseudochlorella signiensis*	SAG 2300	Africa
**LT560360**	*Pseudochlorella signiensis*	SAG 7.90	Antarctica: South Orkney Islands, Signy Island, soil
**HQ292734**	*Pseudomuriella aurantica*	KF43	Czech Republic: Zbrašovská Cave, cave sediment
**KF673365**	*Pseudomuriella engadinensis*	SAG 221-3	Switzerland: Unterengadin, soil
**AY493587**	*Pseudophormidium* sp.	ANT-LPE.3	Antarctica: Long peninsula, Vestfold Hills, saline Pendant lake, microbial mat
**KF144198**	*Pseudopleurococcus printzii*	SAG 467-1	Switzerland: Rosenau/Basel, bog water
**MH703749**	*Pseudostichococcus monallantoides*	Ru-s-4-2	Germany: Prora, Ruegen, biocrust, sand dunes, Baltic Sea coast
**MT078185**	*Pseudostichococcus monallantoides*	SAG 2067	former Yugoslavia: Dalmatia, Island of Lavsa, soil
**MT078184**	*Pseudostichococcus monallantoides*	SAG 379-4	USA: Massachusetts, aquarium (freshwater)

**KM020066**	*Pseudostichococcus monallantoides*	SAG 380-1	Germany: from a culture of *Enteromorpha compressa* (marine)
**MT078186**	*Pseudostichococcus monallantoides*	UTEX 2249	Laboratory contamination
**DQ821514**	*Pseudothrix borealis*		USA: Amanak Island, Alaska, mid intertidal boulders near the Dutch Harbor airport
**KM20039**	*Pumiliosphaera*		
**KM020039**	*Pumilosphaera acidophila*	SAG 15.91	Italy: Napoli, Pozzuoli solfatara at Pisciarelli, freshwater
**AJ439399**	*Pumilosphaera protothecoides* var. *acidicola*	strain 124	Italy: Pisciarelli, Campania, freshwater, pH 2.0
**AF388378**	*Radiococcus polycoccus*	SAG 217-1c	UK: Botanical Garden University of. Cambridge, freshwater
**AJ311640**	*Raphidonema pyrenoidifera*	CCAP 470/5	Antarctica: Chapman Ridge, soil
**Z47997**	*Rexenia paucicellulare*	SAG 463-1	Switzerland: Basel, Botanical Garden University Basel, Victoria green house, small basin, stagnant water
**Z47998**	*Rexenia sarcinoidea*	UTEX 1710	USA: Texas, Bastrop, Bastrop National Park, soil
**KM268886**	*Roholtiella bashkiriorum*	RU9	Russia: Republic of Bashkortostan, Bolsheustikinskoye, meadow land, soil
**KM268888**	*Roholtiella edaphica*	LG-S11	Russia: Republic of Bashkortostan, forb-grass steppes near Sibay city, soil
**AM087962**	*Sarcinofilum mucosum*	SAG 24.93	USA: Texas, Llano Co., shallow temporary pool in granite rock
**MF034648**	*Sarcinofilum mucosum*	SAG 4.90	Antarctica: Prinzess Elizabeth Land, Vestfold Hills, freshwater
**HG514422**	*Scenedesms rubescens*	SAG 5.95	France: near Bordeaux, from a culture of a brown alga
**AY510466**	*Scenedesmus dissociatus*	UTEX 1537	
**AB055801**	*Scenedesmus littoralis*		
**X74002**	*Scenedesmus rubescens*	CCAP 232/1	
**JN385286**	*Schizothrix arenaria*	HA4233-MV5 clone p5BA	USA: Oahu, Hawaii, Kawai Nui Marsh Nature Preserve, Kailua, mud puddle in a ditch
**MH176132**	*Scotiellopsis reticulata*	FACHB-2317	
**AF334700**	*Scytonema hyalinum*	clone 144-3C + 159-3	USA: Paradise Range, California, soil
**KM020086**	*Spongiochloris excentrica*	SAG 280-1	USA: Tennessee, from soil near Rock Island, Caney Fork river
**KY086473**	*Spongiococcum tetrasporum*	ACSSI 120	USA: Soil from a cotton field
**KM020135**	*Spongiococcum tetrasporum*	SAG 29.95	USA: Alabama, Green Country, soil from cotton field
**FR865740**	*Sporotetras polydermatica*	CCAP 31/5	UK: Windermere, Cumbria, England, freshwater

**AF218371**	*Stenomitos*		
**MH688849**	*Stenomitos* sp.	RU-0-2	Germany: Baabe, Ruegen, biocrust, sand dunes, Baltic Sea coast
**AF218371**	*Stenomitos tremulus*	UTCC 471	Canada: Northwest terretories
**AM412751**	*Stichococcus chloranthus*	UTEX 315	Germany: Bernburg/Anhalt
**GU017650**	*Symbiochloris reticulata*	SAG 53.87	Spain: Teneriffe, Roque Chinotére, phycobiont of lichen *Lobaria pulmonaria* var. *meridionalis*
**GU017646**	*Symbiochloris symbiontica*	SAG 2099	Japan: Geihoku-cho, Hiroshima-Pref. , surface of lichen *Graphis* sp.
**KM020026**	*Tetracystis aplanopsora*	SAG 91.80	Cuba: soil near Havana
**KM020030**	*Tetracystis diplobionticoidea*	SAG 33.95	USA: AZ, Apache Junction, arid soil
**KM020076**	*Tetracystis dissociata*	SAG 207-1	Switzerland: soil from Schweiz Nationalpark
**KM020075**	*Tetracystis illinoisensis*	SAG 93.80	USA: IL, Effingham, from petri dish exposed to air from an automobile
**KM020017**	*Tetracystis pampae*	SAG 22.95	Italy: South Tyrol, Pitschberg, spoil from Dolomites
**JN903997**	*Tetracystis pampae*	SAG 96.80	USA: TX, Pampa, soil from flower-bed
**KM020029**	*Tetracystis sarcinales*	SAG 19.94	former Yugoslavia: soil from Isle of Lavsa
**KM020024**	*Tetracystis tetraspora*	SAG 98.80	Cuba: soil from Havana
**JN968583**	*Tetracystis texensis*	SAG 99.80	USA: Texas, Travis Co., soil from Pilot Knob
**MH703775**	*Tetradesmus arenicola*	SAG 2564	Ukraine: Danube Delta Biosphere Reserve, Kiliya district, Odessa oblast, biocrust, baltic sea coast
**MH703774**	*Tetradesmus arenicola*	WD-1-6	Germany: Warnemünde, biocrust, sand dunes, Baltic sea coast
**AY510468**	*Tetradesmus bajacalifornicus*	BCP-LG2-VF16	USA: Sierra San Pedro Martir of Baja California, Mexico, decomposed granite sandy soil
**HQ246450**	*Tetradesmus bajacalifornicus*	ZA1-7	South Africa: 6 K N of Klawer along N7
**AY510474**	*Tetradesmus deserticola*	BCP EM2-VF3	USA: Sierra San Pedro Martir of Baja California, Mexico, decomposed granite sandy soil
**AY510471**	*Tetradesmus deserticola*	BCP-SNI-2	USA: San Nicolas Island, California, soil
**HG514429**	*Tetradesmus distendus*	SAG 2003	Germany: Berlin, Lake Havel (freshwater)
**FR865722**	*Tetradesmus incrassatulus*	CCAP 276/43	Peru: Lake Yanayacu (freshwater)
**FR865731**	*Tetradesmus obliquus*	CCAP 276/52	New Zeeland
**MG022741**	*Tetradesmus obliquus*	276/3C	Germany: Marburg, freshwater
**FR865726**	*Tetradesmus obliquus*	CCAP 276/49	Finnland: Lake Tuomiojaervi, Jyvaeskylae
**HG514426**	*Tetradesmus raciborskii*	CCAP 276/35	Germany: Lake Steinhuder Meer (freshwater)
**HG514430**	*Tetradesmus wisconsinensis*	SAG 22.81	Peru: Dpto. Ancash, Laguna Pataccocha
**DQ275461**	*Tetrastichococcus jenerensis*	SAG 2138	Malaysia: Kelantan, Kampong Kuala Jenera, surface soil crust on unidentified tree base in a secondary lowland rainforest, 400m a.s.l.

**JQ083654**	*Tolypothrix campylonemoides*	FI5-MK38 clone p10D	USA: Fort Irwin NTC, San Bernadino Co, California, sand
**GQ287651**	*Tolypothrix distorta*	SAG 93.79	Spain: Mallorca, from a south-exposed wall of porous stones
**JQ083651**	*Tolypothrix tenuis* f. *terrestris*	UFS-BI-NPMV-1A2-F06 clone p13E	USA: Utah, foothills of the Onaquee Mountains, arid soil after a burn
**EU123942**	*Trebouxia aggregata*	SAG 219-1d	phycobiont of lichen *Xanthoria parientina*
**Z21553**	*Trebouxia asymmetrica*	SAG 48.88	Spain: Catalunya, Sa. de Roses, roadside at the street to El Port de la Selva, phycobiont of lichen *Diploschistes albescens*
**EF429297**	*Trichocoleus badius*	CRS-1	USA
**KF307604**	*Trichocoleus desertorum*	ATA4-8-CV2 clone ACE	Chile: biocrust in Atacama Desert, desert soil
**MT078171**	*Tritostichococcus solitus*	SAG 2406	Germany: tufa-forming karstwater stream "Deinschwanger Bach", western margin of the Franconian Alb, biofilm on rock surface
**MF034653**	*Ulothrix zonata*	SAG 38.86	
**AY278217**	*Ulothrix zonata*	UTEX 745	USA: Lake Michigan (freshwater)
**AY303586**	*Ulva californica*	FH-3.2	USA: San Juan Island, Washington, drift, False Bay
**HQ219374**	uncultured Chlorophyta clone	AY2009A8	France: Lake Pavin or Lake Aydat (freshwater)
**AB058345**	unicellular ulvophyte	MBIC10446	
**AY476821**	*Urospora neglecta*	Seward 8	USA: Seward 8, Alaska, marine
**AY653741**	*Urospora neglecta*	Un155	USA: Seppings I., Barkley, South Dakota
**AY476809**	*Urospora pencilliformis*	Boiler Bay	USA: Oregon, Boiler Bay
**AY476808**	*Urospora pencilliformis*	strain Point No Point	Canada: Point No Point, Vancouver, British Columbia
**AY476819**	*Urospora wormskioldii*	Park 1	Canada: Park 1, Halifax, Nova Scotia
**AY476816**	*Urospora wormskioldii*	strain Louisbourg 5	Canada: Louisbourg 5, Nova Scotia
**FM958481**	*Viridiella fridericana*	SAG 10.92	Italy: Campania, at Mefite di Ansanto, soil from sulphurous vents
**AJ439401**	*Viridiella fridericana*	strain 237	Italy: Mefite di Ansanto, Campania, freshwater, pH 2.0
**MH499915**	*Watanabea acidophila*	ISBAL-1011	Czech Republic: Sokolov mining district (Chodov), acidic soil-coal clays

**MH499916**	*Watanabea acidophila*	ISBAL-1112	Czech Republic: Sokolov mining district (Chodov), dump after coal mining, acidic soil- volcanic ashes (pH 3.5)
**MH499914**	*Watanabea alpicola*	FG2-10/3	Germany: forest soil
**MH499913**	*Watanabea alpicola*	SAG 2580	Switzerland: Unterengadin, soil
**MH499912**	*Watanabea patagonica*	CCAP 6091/1	Argentinia: Patagonia, Acid Lake Caviahue
**FM958480**	*Watanabea reniformis*	SAG 211-9b	UK: West Humble, Dorking, garden basin
